# Distinct metabolic states guide maturation of inflammatory and tolerogenic dendritic cells

**DOI:** 10.1038/s41467-022-32849-1

**Published:** 2022-09-02

**Authors:** Juraj Adamik, Paul V. Munson, Felix J. Hartmann, Alexis J. Combes, Philippe Pierre, Matthew F. Krummel, Sean C. Bendall, Rafael J. Argüello, Lisa H. Butterfield

**Affiliations:** 1 Parker Institute for Cancer Immunotherapy, San Francisco, CA 94129 USA; 2 Systems Immunology and Single-Cell Biology, German Cancer Research Center (DKFZ), Heidelberg, Germany; 3 Department of Pathology, University of California San Francisco, San Francisco, CA USA; 4 ImmunoX Initiative, University of California San Francisco, San Francisco, CA USA; 5 CoLabs, University of California San Francisco, San Francisco, CA USA; 6 Aix Marseille Univ, CNRS, INSERM, CIML, Centre d’Immunologie de Marseille-Luminy, Marseille, France; 7 Institute for Research in Biomedicine (iBiMED) and Ilidio Pinho Foundation, Department of Medical Sciences, University of Aveiro, 3810-193 Aveiro, Portugal; 8 Shanghai Institute of Immunology, Department of Microbiology and Immunology, Shanghai Jiao Tong University School of Medicine, Shanghai, 200025 PR China; 9 Department of Pathology, Stanford University, Palo Alto, CA 94304 USA; 10 Department of Microbiology and Immunology, University of California San Francisco, San Francisco, CA USA

**Keywords:** Translational immunology, Cell biology

## Abstract

Cellular metabolism underpins immune cell functionality, yet our understanding of metabolic influences in human dendritic cell biology and their ability to orchestrate immune responses is poorly developed. Here, we map single-cell metabolic states and immune profiles of inflammatory and tolerogenic monocytic dendritic cells using recently developed multiparametric approaches. Single-cell metabolic pathway activation scores reveal simultaneous engagement of multiple metabolic pathways in distinct monocytic dendritic cell differentiation stages. GM-CSF/IL4-induce rapid reprogramming of glycolytic monocytes and transient co-activation of mitochondrial pathways followed by TLR4-dependent maturation of dendritic cells. Skewing of the mTOR:AMPK phosphorylation balance and upregulation of OXPHOS, glycolytic and fatty acid oxidation metabolism underpin metabolic hyperactivity and an immunosuppressive phenotype of tolerogenic dendritic cells, which exhibit maturation-resistance and a de-differentiated immune phenotype marked by unique immunoregulatory receptor signatures. This single-cell dataset provides important insights into metabolic pathways impacting the immune profiles of human dendritic cells.

## Abstract (web summary)

Assessing metabolic activity within single cells rather than at a population level has a number of advantages. Here, the authors use a flow and mass cytometry based approach that assess the metabolic differences between populations of human immune stimulatory and tolerogenic dendritic cells.

## Introduction

Dendritic cells (DC) bridge innate and adaptive immunity through recognition and processing of pathogen and danger-associated signals for orchestrating cytokine-mediated inflammatory responses and priming antigen-specific T cell activation^1^. DC activation and maturation is a highly coordinated response associated with phenotypic and morphologic changes, which enable functional specialization for mounting protective immunity or tolerance to self-antigens^2^. The DC maturation process results in upregulation of major histocompatibility complexes (MHC), costimulatory molecules (CD86, CD80, CD40, ICOSL) trafficking receptors (CCR7) and secretion of proinflammatory cytokines^3^. Emerging research has identified adaptations in cellular metabolism that are central to accommodate energy demands associated with functional changes in transcriptional and biosynthetic pathways necessary for DC survival, migration and effective T cell priming capacity^4^.

Similar to the Warburg effect in cancer cells, a profound shift from oxidative phosphorylation (OXPHOS) to aerobic glycolysis upon Toll-like receptor (TLR) activation was shown to be central metabolic rewiring in murine bone marrow-derived DCs (BMDCs)^5^. This immediate-early glycolytic increase within minutes of TLR-stimulus, is controlled by the TBK1-IKKε-Akt signaling axis to activate the rate-limiting glycolytic enzyme hexokinase 2 (HK2), which is essential for supporting de novo synthesis of fatty acids for ER and Golgi expansion^6^. While TLR-activated DCs become more dependent on extracellular glucose, it was demonstrated that intracellular glycogen stores support the early glycolytic flux and immune functions^7^. While the early stages of BMDC activation maintained increased OXPHOS, the onset of sustained glycolytic reprogramming induced iNOS-dependent generation of nitric oxide (NO) from arginine, which blocks mitochondrial electron transport and respiration items^8,9^. BMDC switch to glycolysis and lactic acid fermentation as a rapid source of ATP and further engage pentose phosphate pathway (PPP) for increased nucleotide biosynthesis and NADPH for generation of reactive oxygen species (ROS)^10^. Together these complex pathways program murine DC’s ability to process and present antigens for proper activation of adaptive immune branches.

While glycolytic metabolism is a hallmark of murine BMDC activation, this phenomenon does not directly translate to human DC^10–13^ and recent evidence suggests that context-specific metabolic reprogramming governs changes in immature, steady-state, inflammatory activation and initiation of immune tolerance in different microenvironmental and pathophysiological settings^4,13^. Furthermore, diverse metabolic programs and mitochondrial reprogramming underlie cellular fate and function of distinct DC subtypes^14^. Metabolic differences associated with deregulated OXPHOS, glycolysis and fatty acid oxidation (FAO) programs were also shown to influence anti-inflammatory phenotype of tolerogenic DCs (tol-DC)^15^, which maintain immune tolerance by inhibiting effector and autoreactive T cells, and polarizing development of regulatory T cell (Treg) responses^16^.

The mammalian target of rapamycin (mTOR) and AMP-activated protein kinase (AMPK) are critical signaling factors regulating cellular metabolism, but their regulation in the context of human monocyte-derived development is not well understood. As a downstream target of the PI3k/Akt pathway, mTOR is an important upstream activator of glycolytic reprogramming driving high metabolic demands of TLR-activated murine macrophages and DCs^17^. As a critical cellular nutrient sensor controlling an array of cellular responses, growth and survival, mTOR concurrently supports de novo biosynthesis of lipids, proteins, and amino acids^11,18^. Activation of AMPK opposes mTOR dependent glycolytic reprogramming, skewing cellular metabolism towards energy conservation driving mitochondrial biogenesis via peroxisome proliferator-activated receptor-γ (PPARγ) co-activator-1α (PGC1α) signaling axis to increase activity of mitochondrial enzymes and OXPHOS. AMPK also upregulates carnitine palmitoyltransferase 1α (CPT1α) favoring catabolic FAO^11,18,19^. The PI3K/Akt/mTOR pathway^20^ along with the p38 MAPK, ERK1/2 and STAT3 signaling axis^21^ have been implicated in controlling glycolytic phenotypes of the tol-DC, but the role for AMPK in both immunologic and tol-DC biology is largely understudied, and the precise role of mTOR/AMPK balance is controversial^4,13^.

While providing invaluable insights to the field of immuno-metabolism, the technical limitations of bulk cellular measurements often used to measure metabolic respiration by means of metabolite tracing and/or oxygen consumption (OCR) and extracellular flux analyses (ECAR), are not able to adequately capture the newly-appreciated phenotypic and functional and diversity associated with the heterogeneous nature of in vitro DC culture systems^22,23^. As key regulators of immune homeostasis, monocytic DC have been critical resource for diverse cell therapy applications including priming anti-tumor T-cell responses as cancer vaccines^24^, or in the opposing role as tolerogenic promoting immune suppression for organ transplantation and autoimmune disease treatment^25^.

Emergence of single-cells approaches using RNA sequencing and high-dimensional mass (cytometry by time of flight, CyTOF) and fluorescent cytometry-based techniques paralleled with histology-based multiplexed imaging (MIBI-TOF) enables robust estimation of immuno-metabolic states of individual cells in the context of heterogeneous cell populations as well as spatial organization in tissues^26–30^.

In this study, we coupled single-cell energetic metabolism by profiling translation inhibition (SCENITH)^27^ and CyTOF-based single-cell metabolic regulome profiling (scMEP)^29^ to integrate functional measurements with quantifying metabolite transporters and enzymes across major cellular metabolic axes. We show previously obscured coordinate activation of multiple metabolic pathways along distinct stages of monocytic DC differentiation and maturation. Our mapping of functional metabolic states and the underlying metabolic protein regulome further showed that elevated mTOR:AMPK phosphorylation ratio with upregulation of OXPHOS, glycolytic and fatty acid oxidation metabolism underlies the metabolic hyperactivity of the immunosuppressive phenotype of tolerogenic DC.

## Results

### Immuno-metabolic profiling of monocyte to DC differentiation reveals extensive reprogramming from glycolytic to predominantly mitochondrial metabolism

**Fig. 1:**
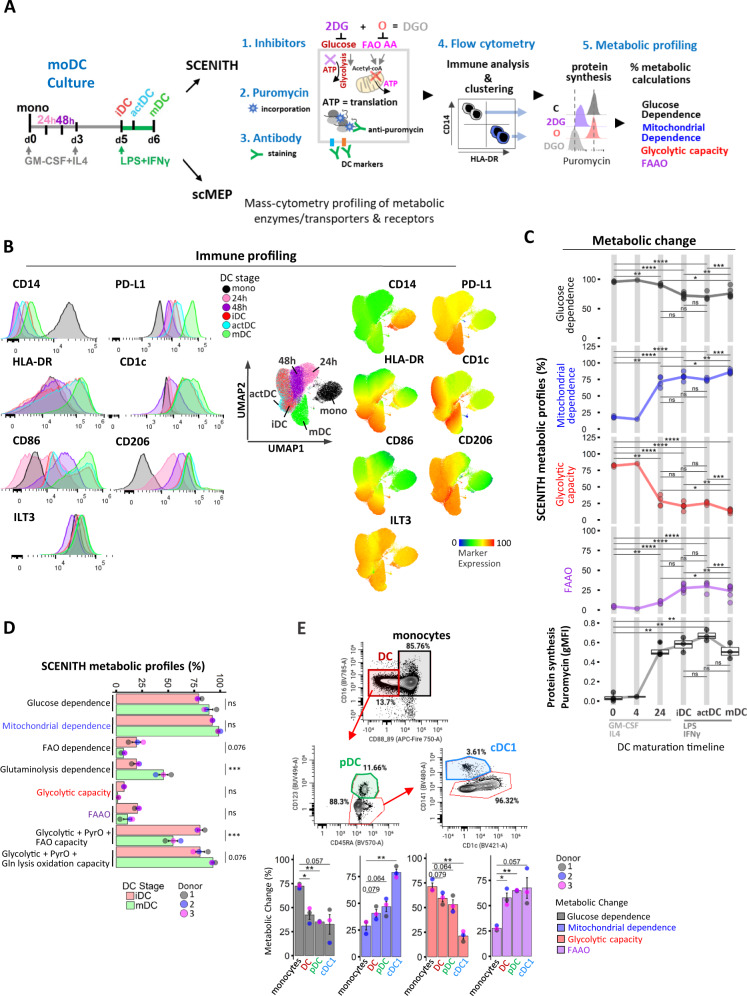
Distinct metabolic profiles regulate in vitro DC-lineage differentiation and blood DC. **A** Conceptual overview of in vitro culture conditions and experimental setup for scMEP and SCENITH functional metabolic profiling and immune characterization of DC differentiation states. **B** Expression of immune markers over the course of DC generation is illustrated in flow-cytometry histograms. UMAP clustering based on DC immune markers (CD14, HLA-DR, CD86, CD206, CD1c, PD-L1, ILT3, CD11c, CD276) across DC differentiation stages. Heatmap overlays depict immune marker expression. **C** Overview of kinetic changes in percentual SCENITH parameters and protein synthesis measurements across DC differentiation timeline. Lines represent average SCENITH profiles (precursor stages 0 h and 24 h represent three independent donors), iDC and mDC represent six independent donors, precursor 4 h time point is only a reference point for visualization purposes and represents 1 donor. **D** Percentual SCENITH comparisons between iDC and mDC including Etomoxir and CD-839-derived parameters are shown (bar graphs represent 3 independent replicates from 1 donor with mean ± SE). PyrO abbreviates proteins synthesis due to pyruvate oxidation. **E** Shown are gating strategies for immune characterization and percentual SCENITH profiles for freshly isolated blood monocytes and DC populations from 3 independent donors with mean ± SE. Statistical significance in **A**, **E** was calculated via one-way ANOVA with Tukey’s *post-hoc* test, **D** using two-sided Student’s *t*-test. For all panels, *P*-values are represented as *p ≤ 0.05, **p ≤ 0.01, ***p ≤ 0.001, ****p ≤ 0.0001. *p*-values < 0.05 were considered statistically significant (ns). Box plots indicate second and third quantile (box), median (horizontal line) and 1.5× the interquartile range (whiskers). Source data are provided as a source data file.

To evaluate the impact of metabolic pathway inhibition during monocytic DC differentiation, we employed SCENITH coupled with a multi-parametric panel encompassing DC surface and signaling markers (Fig. 1A). This enabled us to employ both manual gating and unsupervised clustering approaches to profile immune-phenotypes and metabolic activity of CD14 + monocytes, DC precursors (mono 24 h/48 h), immature DC (day 5 iDC), DC after 4 h LPS + IFN-γ (actDC), and DC after 24 h of LPS + IFN-γ'' (mDC) at single-cell level. Dimensionality reduction based on nine immune markers identified 5 distinct clusters of differentiation states with iDC and actDC co-occupying the same cluster (Fig. 1B). Monocyte differentiation induced rapid loss of CD14, which was paralleled by maturation upregulation of MHC receptor HLA-DR, co-stimulatory molecules CD86, CD206, including acquisition of the conventional DC 2 (cDC2) marker CD1c (BDCA-1), checkpoint regulator programmed cell death ligand-1 (PD-L1/CD274) (Fig. 1B) and modest increase in co-inhibitory Ig-like transcript 3 (ILT3/CD85) (Fig. 1B). The DC SCENITH panel and gating strategies for precursors and DC populations are shown in Supplementary information (Supplementary Table 1, Supplementary. Fig. 1A).

In agreement with Argüello et al.^27^, monocytes relied primarily on glucose oxidation having the highest glycolytic capacity and minimal dependency on mitochondrial energy production (Fig. 1C). Within 24 h of GM-CSF/IL4 stimulus, monocytes precursors increased mitochondrial dependence from 18% to 72%, which was accompanied with decreased glycolytic capacity from 82% to 28%. Day 5 iDC exhibited further increase in mitochondrial dependence to 79% along with increased protein synthesis which peaked 4 h post LPS/IFNγ-induced DC maturation along with transient increase in glycolytic capacity in actDC (Fig. 1C). Fully matured mDC exhibited lowest protein synthesis, and 86% mitochondrial dependence with moderate FAAO capacity (24%) and low glycolytic capacity (14%) (Fig. 1C). Etomoxir and CB-839 inhibitors were used to further separate contributions of fatty acids (long-chain) and glutamine, respectively, towards fueling protein synthesis in iDC and mDC. These inhibitors did not alter DC markers’ expression and allowed us to reveal that while iDC showed similar 19% Glutaminolysis and FAO dependence, mDC had lower, 7% FAO dependency and increased 41% Glutaminolysis dependence (*P* < 0.05; Fig. 1D, Supplementary Fig. 1B, C).

To determine if the metabolic changes observed during differentiation of cells in cell culture media in-vitro correlate with ex-vivo data, we derived SCENITH parameters for monocytes and CD123^+^ plasmacytoid (pDC) and CD141^+^CD1c^-^ cDC1 sub-populations in freshly isolated PBMCs (Fig. 1E). Similar to in vitro conditions, ex vivo monocytes had highest glucose dependence (72%) and 71% glycolytic capacity. In contrast, cDC1 exhibited highest mitochondrial dependence (79%) and unlike inflammatory in vitro matured DC, both cDC1 and pDC exhibited highest FAAO capacity (65%). This suggests that high levels of glucose in culture media is potentially skewing a preference for glucose as an energy source for in vitro derived DC.

### Temporal changes in the metabolic regulome reflect functional reprogramming of mitochondrial respiration and axillary pathway activation in DC

**Fig. 2:**
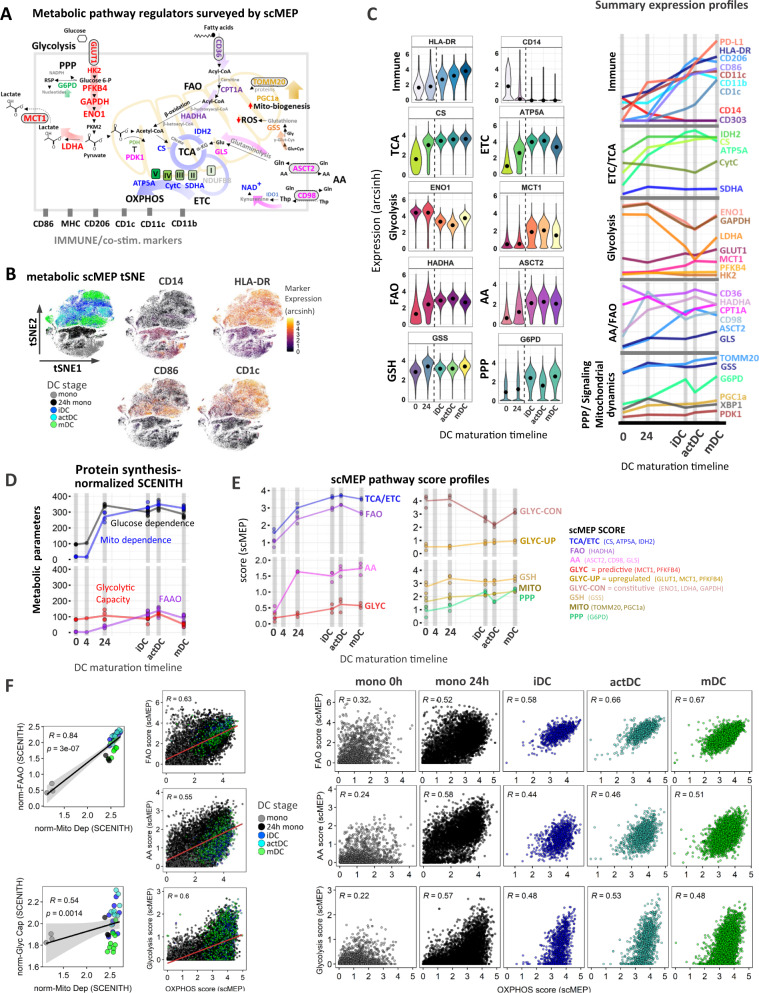
Dynamic changes in metabolic regulome and co-expression of multiple metabolic pathways governs the immune reprogramming of DC. **A** Graphical overview of the scMEP approach depicting metabolic enzymes, signaling factors and metabolite receptors spanning multiple metabolic pathways as well DC-lineage markers profiled by CyTOF. **B** tSNE visualization of DC stages using scMEP metabolic markers. Heatmap overlay of single-cell CD14, HLA-DR, CD86 and CD1c (arcsinh transformed) expression highlights associations between DC stages and immune marker expression. Data show one representative experiment (out of *N* = 3 donors). **C** Shown are (arcsinh transformed) expression values for selected scMEP immune markers across DC differentiation states. Black dots represent population medians and the dotted line separates early precursors from iDC, actDC, and mDC stages. Violin plots are representative of 1 donor (out of *N* = 3). On the right side are represented summary (mean) kinetic expression profiles for all measured immune and metabolic scMEP parameters across DC differentiation. **D** Kinetic profiles of protein synthesis-adjusted SCENITH parameters (calculated as described in materials and methods) to obtain metabolic pathway-dependent changes accounting for ATP production. Lines highlight mean SCENITH profiles (precursor stages represent 3 independent donors, iDC, actDC, and mDC represent six independent donors). **E** Kinetic profiles for calculated mean scMEP pathways scores are illustrated. Connecting lines visualize mean pathway changes (*N* = 3 donors). **F** Correlations between median SCENITH parameters and respective calculated median scMEP pathway scores with Spearman correlation coefficient (R), *p*-value and grey shading denoting 95% confidence interval (CI). Middle and multi-panel graphs depict single-cell scMEP scores for combined and individual DC sample time points respectively. Subsampled single-cell data points for the individual donor (out of *N* = 3) are shown. Source data are provided as a Source Data file.

To further map the metabolic rewiring underlying functional specialization of antigen presenting DC we utilized scMEP to quantify the expression of phenotypic markers in conjunction with rate-limiting metabolic enzymes, metabolite transporters and signaling factors encompassing several metabolic pathways depicted in Fig. 2A. Kinetic profiles for multiple DC-lineage surface markers recapitulated SCENITH immune-profiling. Along with the loss of CD14, there was a maturation-specific upregulation in HLA-DR, CD86, PD-L1 and CD1c, while CD206, CD11c, CD11b peaked in actDC (Fig. 2B, C, Supplementary Fig. 2A). Dimensionality reduction of scMEP metabolic parameters using tSNE maps enabled visual separation of DC differentiation states as characterized by immune marker overly expression (Fig. 2B). The increase in mitochondrial dependence measured by SCENITH was complemented by scMEP, in which GM-CSF/IL4 treatment triggered robust upregulation of components of the tricarboxylic acid (TCA) cycle (IDH2, CS) and electron transport chain (ETC) complexes (SDHA, ATP5A) (Fig. 2C, Supplementary Fig. 2B). Glycolytic enzymes ENO1, GAPDH and LDHA were highly expressed in monocytes and their expression decreased following differentiation, which consistent with the reduced glycolytic capacity of maturing DC (Figs. 1C, 2C). However, we also observed that Glucose and lactate transporters GLUT1 and MCT1 respectively, together with low expression levels of PFKFB4 were upregulated during DC differentiation (Fig. 2C, Supplementary Fig. 2B). scMEP identified concurrent upregulation of metabolic markers regulating fatty acid oxidation (HADHA) together with AA transporters (ASCT2, CD98) and glutaminase (GLS). This further complements the Etomoxir and CD-839 SCENITH inhibitor data, and suggests that in addition to glucose, DC also utilize fatty acids and AA over the course of differentiation (Fig. 2C, Supplementary Fig. 1C). Of note FA transporter CD36 and enzyme CPT1A exhibited moderate decrease followed by constitutive expression across maturation. Intracellular glutathione redox status plays important role in differentiation and Th1 and Th2 responses of DCs^31,32^. Glutathione synthase (GSS) functions in glutathione (GSH) biosynthesis to protect cells from oxidative damage^33^. GSS exhibited increased expression towards iDC stage and remained constant following DC maturation. The pentose phosphate pathway (PPP) represents a branch of glucose metabolism, which regulates redox homeostasis, production of reactive oxygen species (ROS), nitric oxide (NO) and fatty acid synthesis by producing the vital intermediate NADPH as well as nucleic acid building block ribose 5-phosphate (R5P)^34^. The effects of PPP on DC activation have been only studied in context of murine DC differentiation to date. The study by Everts et al.^6^, showed that inhibition of PPP enzyme glucose-6-phosphae dehydrogenase (G6PD) diminished LPS-mediated proinflammatory cytokine production and lipid accumulation, which resulted in preventing murine BMDC maturation. PPP was elevated in iDC and mDC stages with significant decrease in actDC (Supplementary Fig. 2B). Mitochondrial biogenesis and dynamics were monitored using PPARγ co-activator-1α (PGC1α) and translocase of outer mitochondrial membrane 20 (TOMM20), which were both upregulated through DC differentiation (Fig. 2, Supplementary Fig. 2B). We note that the expression changes of scMEP metabolic enzymes were normalized to the observed 4-fold increase in cell volume from monocyte to mDC.

### scMEP-based co-expression patterns define single-cell DC immune diversity

We integrated the SCENITH functional parameters with scMEP co-expression patterns to calculate metabolic pathway scores across DC differentiation. A limitation in SCENITH calculation output is that it cannot distinguish between wells that have different magnitudes of protein synthesis levels even though the overall metabolism may be different. Because protein translation was demonstrated to be a highly energy-dependent metabolic process and a reliable and highly stable readout of cellular ATP production^27^, it is used as a surrogate readout for inhibitor-induced changes in cellular metabolic ATP output by flow cytometry. To reflect the contribution of each SCENITH pathway percentual measurements with respect to total protein synthesis over time, we multiplied each SCENITH parameter by gMFI of puromycin MFI accounting for the changes in background levels according to the following formula: [(SCENITH parameter)*(C-DGO)] (Fig. 2D, Supplementary Fig. 3A). We tested correlations between protein synthesis-adjusted SCENITH metabolic profiles and scMEP marker co-expression and used a previously described approach^29^ to derive in silico scores, used to represent metabolic pathway activation. Using scMEP scores we were able to map temporal changes in OXPHOS, glycolysis, FAO, AA, PPP, GSH and mitochondrial dynamics (MITO) remodeling across DC differentiation timeline (Fig. 2E) with statistical significance depicted in Supplementary Fig. 3B. Due to more complex co-expression patterns of glycolytic markers, we also calculated separate scMEP scores for upregulated (GLYC-UP) and constitutive (GLYC-CON) arms of the DC glycolytic pathway (Fig. 2E).

### Temporal changes and engagement of multiple metabolic pathways underlies heterogeneity of DC immune phenotypes

**Fig. 3:**
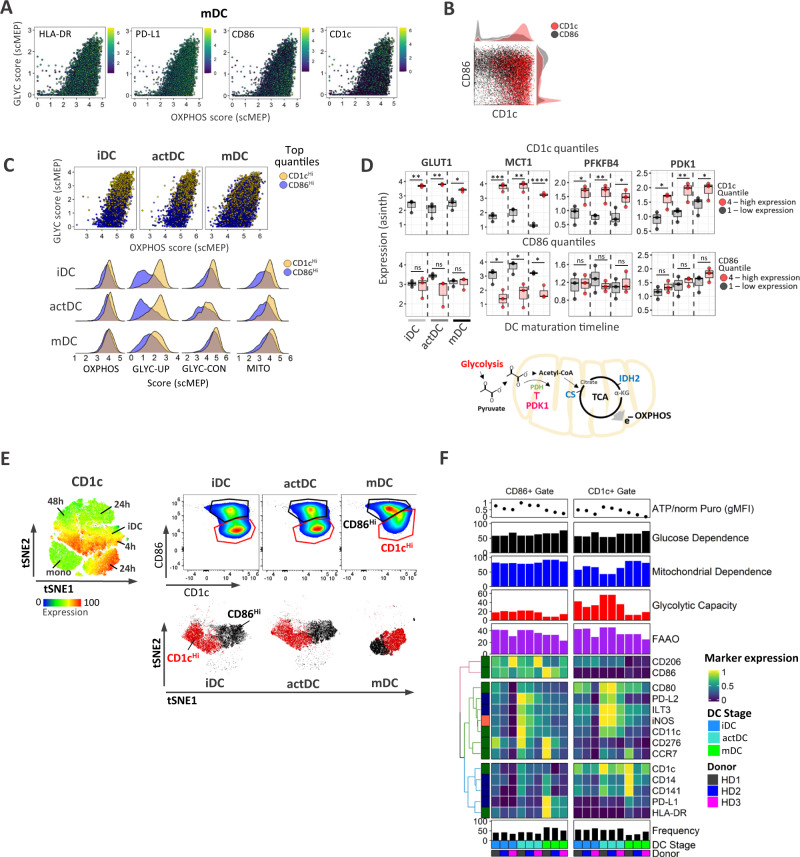
Metabolic heterogeneity associates with phenotypic polarization of CD1c^hi^ and CD86^hi^ DC populations. **A** Correlation analysis for single-cell glycolytic glycolysis and OXHOS scores with heatmap expression single-cell overly of indicated immune markers. Subsampled single-cell data points for the individual donor (out of *N* = 3) are shown for the entire figure. **B** Mass cytometry scatter plots for CD1c and CD86 expression profiles were used to emphasize the distribution of CD1c^hi^ and CD86^hi^ populations. **C** Shown are single-cell scatter plot comparisons of the top 4th quantiles from CD1c^hi^ (blue) and CD86^hi^ (gold) DC populations. Lower graphs represent histogram distributions of single-cell scMEP metabolic pathway scores in CD1c^hi^ and CD86^hi^ populations. **D** Box plots represent median expression values of glycolytic enzymes and PDK1 in the 1st (lowest, black) and 4th (highest, red) quantile from CD1c and CD86 populations across iDC, actDC, and mDC from 3 independent donors. Statistical significance was calculated using two-sided Student’s *t*-test. A role for PDK1 in pyruvate to Acetyl-CoA conversion is depicted underneath the graphs. **E** tSNE visualization of SCENITH profiling depicts clustering of DC stages with CD1c expression heatmap overlay. Adjacent gating strategy was used to select CD1c^hi^ and CD86^hi^ populations, whose spatial distribution is emphasized (with matching colors) on tSNE maps divided into separate iDC, actDC, and mDC stages. **F** Heatmap of gMFI expression for collection of SCENITH phenotyping markers in CD1c^hi^ and CD86^hi^ populations from iDC, actDC, and mDC (*N* = 3 donors). Donor label, DC differentiation stages population frequency, protein synthesis levels along with SCENITH percentual metabolic profiles are annotated. For all panels, *P*-values are represented as *p ≤ 0.05, **p ≤ 0.01, ***p ≤ 0.001, ****p ≤ 0.0001. *p*-values < 0.05 were considered statistically significant (ns). Box plots indicate second and third quantile (box), median (horizontal line) and 1.5× the interquartile range (whiskers). Source data are provided as a Source Data file.

Collectively, metabolic changes in SCENITH and scMEP pathway analyses suggested simultaneous increase of mitochondrial and glycolytic pathways at specific stages of DC differentiation (Fig. 2D, E). Because the SCENITH profiling used to derive scMEP scores represents a bulk metabolic measurement encompassing whole well cultures, it remained unclear whether there are distinct OXPHOS and/or glycolytic DC sub-populations contributing to the final metabolic output. Independently calculated single-cell scMEP scores revealed that differentiating DC populations simultaneously upregulate both OXPHOS and FAO pathways with homogenous distribution throughout differentiation with peak expression at the iDC and actDC activation stages (Fig. 2F). The AA metabolism followed a similar but less correlative upregulation pattern as FAO and exhibited the highest heterogeneity in mDC (Fig. 2F). Positive correlations and unified distribution with mitochondrial dependence were also observed for GSH pathway and mitochondrial dynamics scores arguing for the importance of glutathione redox status and mitochondrial biosynthesis during DC maturation (Supplementary Fig. 3C). Coordinate engagement of both OXPHOS and glycolysis, which resembled metabolic remodeling of activated CD8 T cells^26,29^ was observed. However, our single-cell approach revealed that cells span a wide spectrum of possible glycolytic and respiratory scMEP scores, indicating a range of metabolic heterogeneity within each differentiation time point (Fig. 2F). To better capture this previously unrecognized metabolic diversity, we mapped heatmap overlays of DC-lineage marker expression on the single-cell scMEP pathway score co-expression plots. Analysis of mDC revealed that a range of OXPHOS and glycolytic co-expression profiles underlies distinct DC immune phenotypes. Specifically, we saw that while HLA-DR and PD-L1 distribution showed wide glycolytic and OXPHOS potential, CD1c^Hi^ cells exhibited the highest OXPHOS and glycolytic phenotype and the CD86^Hi^ populations were preferentially skewed toward OXPHOS (Fig. 3A).

### Differential coactivation of mitochondrial vs glycolytic pathways underlies DC immune phenotypes and separates CD1c^Hi^ CD86^Hi^ DC populations

To further determine metabolic properties associated with CD1c^+^ vs CD86^+^ phenotypic polarization (Fig. 3B), we divided our single-cell data sets into 4 quantiles, which were determined from CD1c and CD86 expression ranges (Supplementary Fig. 4A). Quantile visualization of single-cell correlation scMEP scores further confirmed that while CD1c^hi^ phenotype associates with both glycolytic and OXPHOS pathways, CD86^+^ polarization skews predominantly towards aerobic OXPHOS metabolism (Supplementary Fig. 4B). Apart from slightly enhanced mitochondrial dynamics, distribution of scMEP population scores for constitutive glycolytic enzymes or additional pathways did not show significant changes between the top CD1c^hi^ and CD86^hi^ quantiles (Fig. 3C, Supplementary Fig. 4C). Additional analysis of differential scMEP marker expression between the CD1c^hi^ (quantile 4) vs CD1c^low^ (quantile 1) cells confirmed that inducible factors GLUT1, MCT1 and PFKFB4 are significantly elevated in CD1c^hi^ populations (Fig. 3D, Supplementary Fig. 4D). Expression of these molecules was significantly downregulated (MCT1) or unchanged (GLUT1, PFKFB4) in CD86^hi^ vs CD86^low^ populations. PDK1 was also significantly increased in the (CD1c^hi^) quantile 4, which was not observed in CD86 quantiles comparisons (Fig. 3D, Supplementary Fig. 4D). PDK1 participates in inhibiting phosphorylation of the pyruvate dehydrogenase complex, thereby preventing conversion of pyruvate produced by glycolysis to acetyl-CoA and its entry to the TCA cycle, as diagramed in Fig. 3D^35^. Its critical role in glucose homeostasis was demonstrated in a study by Tan et al.^36^ in which PDK1-knockdown reduced glycolysis, glucose oxidation and enhanced mitochondrial respiration causing attenuated inflammatory response in M1 macrophages. Therefore, we postulate that elevated PDK1 prevents pyruvate entry into mitochondria and supports the increased glycolytic capacity of CD1c^hi^ DC. To functionally validate our phenotypic mass cytometry data, we analyzed SCENITH parameters of manually gated CD1c^hi^ and CD86^hi^ populations (Fig. 3E). Indeed, CD86^hi^ iDC exhibited high mitochondrial dependence (81%), which increased in the mDC stage reaching 90% together with low glycolytic capacity (10%). In contrast, CD1c^hi^ iDC showed lower mitochondrial dependence (65%) with 35% glycolytic capacity (Fig. 3F). While glycolysis of CD1c^hi^ cells reaches maximal glycolytic capacity in actDC (50%), we observed convergence of the metabolic pathways towards predominantly mitochondrial respiration as cells acquire both immune markers and advance in maturation (Fig. 3C, E). Immune molecules HLA-DR, CD206, PD-L1, CD276 (B7-H3), CCR7 and CD11c were elevated on CD86^hi^, while CD80 and ILT3 were enriched on CD1c^hi^ populations (Fig. 3F, Supplementary Fig. 4F).

### A proportional shift from glucose dependence to FAAO utilization and simultaneous upregulation of several metabolic pathways underly immune-suppressive phenotypes tol-DC

**Fig. 4:**
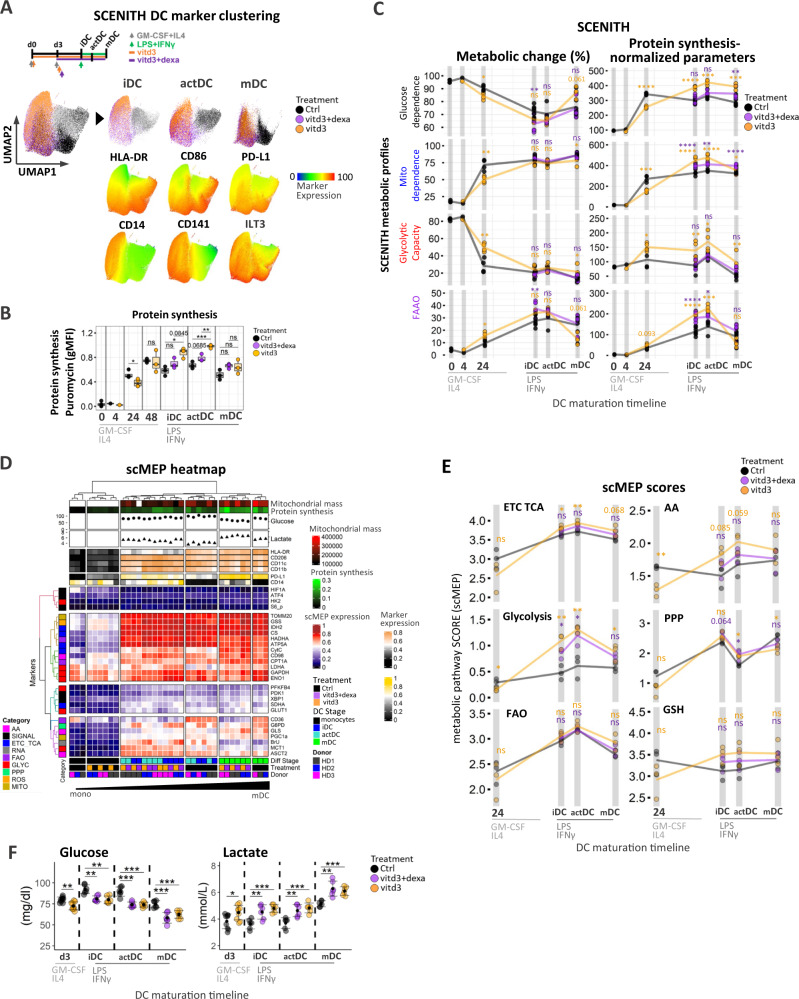
Vitd3 and dexamethasone alters metabolic and signaling networks in immune-suppressive phenotypes of tol-DC. **A** Schematic diagram of tolerogenic DC treatment conditions. Control (black), vitd3+dexa (purple) and vitd3 (orange) cells sampled at iDC, actDC, and mDC were subjected to dimensionality reduction using UMAP. Single-cell heatmaps were overlayed on concatenated (iDC, actDC, and mDC) samples to depict immune marker expression changes between maturation stages in control and tolerogenic cell clusters. **B** Boxplots represent changes in SCENITH puromycin protein synthesis (gMFI puromycin) levels across DC stages and treatment conditions (*N* = 3 donors). **C** Overview of kinetic changes and differences in percentual (left panel) and protein synthesis-adjusted (right panel) SCENITH metabolic parameters between control, vitd3+dexa (purple) and vitd3-treated (orange) DC across differentiation timeline. Connecting lines visualize average pathway changes (precursor stages represent 3 independent donors, iDC, actDC, and mDC represent SIX independent donors). Statistical significance of pairwise comparisons between control and vitd3-dexa-tol (purple asterisk) and control and vitd3-tol samples (orange asterisk) analyses are depicted. **D** Integrated clustering heatmap of DC activation stages based on median arcsinh transformed expression values for scMEP metabolic regulators (*N* = 3). Bottom heatmap annotations include donor labels, treatment conditions and DC differentiation stages. Fluorescent quantitation of mitochondrial size (Mitotracker Deep Red) along with protein translation/ATP levels are annotated in the form of a heatmap. Point annotations representing lactate and glucose supernatant measurements were determined in iDC, actDC, and mDC. Heatmap annotation for DC immune signatures are located at the top of the clustering matrix. scMEP markers are colored according to their metabolic pathway activity. **E** Kinetic profiles for calculated median scMEP pathways scores for control (black), vitd3+dexa (purple) and vitd3 (orange)-treated DC across DC maturation timeline. Connecting lines visualize mean pathway changes (*N* = 3). Statistical significance of pairwise comparisons between control and vitd3-dexa-tol (purple asterisk) and control and vitd3-tol samples (orange asterisk) analyses are depicted. **F** Glucose and lactate measurements in control and tolerogenic DC culture supernatants are shown. Of note glucose level measurement increase in the media between d3 and iDC stage is due to media change at day 3. Three technical replicates from three donors are presented with error bars indicating standard deviation. Multiple comparisons statistical significance in **B**, **C**, **E**, **F** was calculated via one-way ANOVA with Tukey’s *post-hoc* test. For all panels, *P*-values are represented as *p ≤ 0.05, **p ≤ 0.01, ***p ≤ 0.001, ****p ≤ 0.0001. *p*-values < 0.05 were considered statistically significant (ns). Box plots indicate second and third quantile (box), median (horizontal line) and 1.5× the interquartile range (whiskers). Source data are provided as a Source Data file.

**Fig. 5:**
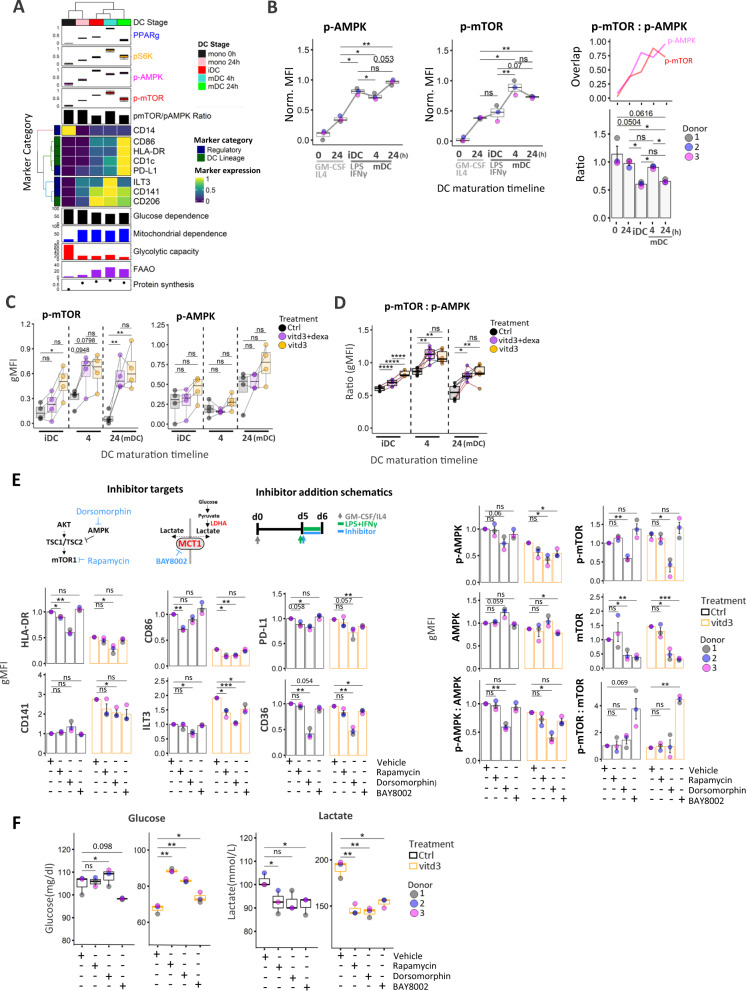
AMPK:mTOR signaling axis and lactate transporter MCT1 are critical regulators of tolerogenic DC. **A** Heatmap and clustering analysis of gMFI expression profiles for DC-lineage markers over time. Average expression from three donors is represented. Protein synthesis levels, Percentual SCENITH parameters, donor label, and DC differentiation stages are annotated along with expression profiles for signaling factors and calculated mTOR:AMPK phosphorylation ratio. **B** Kinetic phosphorylation levels for p-AMPK and p-mTOR across DC differentiation and their trajectory overlaps are depicted. Barplots with mean ± SE represent changes in calculated p-mTOR:p-AMPK ratios (*N* = 3). **C** gMFI expression values of p-AMPK and p-mTOR in control (black), vitd3+dexa (purple) and vitd3 (orange)-treated DC across maturation stages are shown (*N* = 4). Lines connect data points from an individual donor. **D** Boxplots represent changes in calculated p-mTOR:p-AMPK ratios between control (black), vitd3+dexa (purple) and vitd3 (orange)-treated DC (*N* = 5 donors) across maturation timeline. Lines connect data points from an individual donor. **E** Bar graphs with mean ± SE represent gMFI expression values and **F** box plots represent Glucose and lactate measurements for control and vitd3-tol mDC treated with Vehicle (DMSO), Rapamycin (1 μM), Dorsomorphin (3.75 μM) and BAY8002 (80 μM) (*N* = 3). Diagrams of pathway inhibitor targets and timeline for inhibitor treatment are depicted. Multiple comparisons statistical significance in **B**–**F** was calculated via one-way ANOVA with Tukey’s *post-hoc* test. For all panels, *P*-values are represented as *p ≤ 0.05, **p ≤ 0.01, ***p ≤ 0.001, ****P ≤ 0.0001. *p*-values < 0.05 were considered statistically significant (ns). Box plots indicate second and third quantile (box), median (horizontal line) and 1.5× the interquartile range (whiskers). Source data are provided as a Source Data file.

We next applied single-cell approaches to investigate DC skewed to be tolerogenic to identify critical metabolic features and potential biomarkers defining the major modes of in vitro tolerogenic DC generation. Tol-DC were generated using 1α,25-dihydroxyvitamin D_3_ (vitd3) alone (vitd3-tolDC) or in sequential combination with dexamethasone (vitd3-dexa-tolDC) as depicted in Fig. 4A and their immuno-metabolic profiles were monitored along with inflammatory DC across the maturation timeline. Tol-DCs exhibited classical changes with elongated spindle-like characteristics^37^ along with reduced HLA-DR and CD86 with retention of CD14 surface expression, respectively (Supplementary Fig. 5A) with statistical analysis denoted in SupplementarySupplementary. Fig. 5C. HLA-DR^+^CD86^+^ populations were used for all downstream analyses to ensure that our comparisons are representative of DC-cell linages and not undifferentiated CD14 + monocyte contaminations.

Differential expression analysis of the full spectrum of SCENITH markers showed that both tolerogenic signals significantly decreased HLA-DR at the iDC stage and reduced maturation increase of numerous costimulatory molecules including HLA-DR, CD86, and CD1c (Supplementary Fig. 5B, C). CD276 (B7-H3) was specifically downregulated in vitd3-tolDC and expression of CD206, CCR7, CD11c and CD80 was overall upregulated but more variable between the tolerogenic conditions. Neutral amino acid transporter CD98 (LAT1) and CD36 exhibited upregulated and downregulated expression pattern in both tolerogenic DC types respectively (Supplementary Fig. 5B). UMAP visualization using SCENITH immune parameters clustered control from tolerogenic samples and separated distinct DC stages with iDC and actDC resembling closest spatial profiles (Fig. 4A). Single-cell heatmap overlay of selected immune markers demonstrated a progressive increase of HLA-DR and CD86 with maturation in control wells, which was reduced in both tolerogenic counterparts. Consistent with previous reports^12,38^, tol-DC exhibited a robust increase in CD14, CD141, and ILT3, and PD-L1 across all maturation stages (Fig. 4A, Supplementary Fig. 5B, C).

Based on SCENITH parameters, vitd3-tol-DC at the iDC and actDC stages significantly increased global rate of protein synthesis (Fig. 4B). Percentual SCENITH profiles revealed a significant decrease in mitochondrial dependence and a 25% increase in glycolytic capacity in the GM-CSF/IL4 24h-vitd3 precursors (Fig. 4C left panel). While the percentual glycolytic reprogramming was not apparent across later maturation stages, we observed a significant shift from glucose dependence with a 15% increase in FAAO capacity in vid3-dexa-tol-iDC. Protein synthesis-adjusted SCENITH parameters revealed a significant transient increase in glucose dependence and glycolytic capacity predominantly in the vitd3- treated tol-DC with mitochondrial dependance and FAAO capacity significantly increased in both types of tol-DC (Fig. 4C right panel, Supplementary Fig. 4B right panel).

In parallel with SCENITH profiling, dynamic changes in scMEP pathways validate metabolic hyper-activation of tol-DC. Unsupervised clustering analysis of median of scMEP metabolic proteins across 3 donors robustly separated inflammatory from tol-DCs while preserving expression differences within each differentiation/maturation stage (Fig. 4D). Differential expression analysis of scMEP markers showed significant and sustained upregulation of TCA/ETC regulators (CS, CytC, SDHA, ATP5A) together with dramatic and transient increase multiple glycolytic markers (LDHA, MCT1, PFKFB4, ENO1) with the highest degree of differences at the iDC and actDC stages of both types of tol-DCs (Supplementary Fig. 5D). Among robustly upregulated factors were components of mitochondrial dynamics (GSS, TOMM20), amino acid transporter CD98 along with signaling mTOR downstream target pS6K, which validates SCENITH panel measurements (Supplementary Fig. 5D). The CD36 (downregulated in tol-DC) was the most robust predictor of tol-DC phenotypes based on single-cell random forest permutation analysis (Supplementary Fig. 5E). Kinetic analysis of scMEP pathway scores further showed the transient nature of metabolic pathway upregulation in both types of vid3-dexa-tol-DC and in a sense agreed with previous study^12^ showing that glycolytic capacity returns close to normal immunogenic DCs levels following long term (24–48 h) LPS treatment of vid3-dexa-tol-DCs (Fig. 4E). Furthermore, we uncover dynamic changes associated with elevated OXPHOS, FAO and AA metabolism, PPP and increase in GSH activation consistent with increased redox state and production of reactive oxygen species by tol-DC^12,37^. Of note, constitutive and inducible glycolytic scMEP pathway scores were upregulated significantly in vitd3-tol-iDC and actDC as compared to immunogenic counterparts (Supplementary Fig. 5F). In agreement with persistent protein synthesis-adjusted increase in glucose dependence (Fig. 4C right panel) and high glycolytic scMEP scores (Fig. 4E) with upregulated lactate transporter MCT1 expression (Supplementary Fig. 5D), we detected decreased glucose levels with parallel increase in secreted lactate in tol-DC culture supernatants (Fig. 4F).

### mTOR and AMPK phosphorylation states reflect mitochondrial vs glycolytic metabolic programs in DC and increased pmTOR:pAMPK ratio impacts metabolic alterations of tol-DCs

To gain additional insights into signaling cascades regulating inflammatory and tolerogenic DC metabolism, we employed antibodies recognizing the total and phosphorylated forms of AMPK (Thr-183/172) and p-mTOR (Ser-2448) (Supplementary Fig. 6A). We also monitored changes in PPARγ and a downstream mTORC1 target ribosomal protein S6 kinase 1 (pS6K) along with immune markers during DC differentiation (Fig. 5A). Both p-mTOR and p-AMPK were modestly upregulated 24 h following GM-CSF/IL4 induction, but p-AMPK continued to increase from 24 h precursor stage towards iDC, while p-mTOR did not significantly change. P-mTOR transiently increased following 4 h LPS/IFNγ, which paralleled transient increase in glycolytic and FAAO capacity (Figs. 1C, 5A). p-AMPK was transiently downregulated and increased in mDC. The opposing phosphorylation states were further reflected in significant increase in p-mTOR:p-AMPK ratio (Fig. 5A) in actDC (Fig. 5B). Expression of PPARγ also exhibited transient expression in actDC, which mirrored a transient increase in FAAO and pS6K levels significantly correlated with p-mTOR (Fig. 5A, Supplementary Fig. 6B). In agreement with previous reports^20^, we demonstrate that p-mTOR is significantly enhanced at distinct DC stages in both tolerogenic conditions while p-AMPK was not significantly altered from controls. The resulting p-mTOR:p-AMPK ratio revealed persistent and significant skewing towards higher p-mTOR dominance in both tolerogenic DC types (Fig. 5D). In addition, pS6K showed similar trend as p-mTOR and iNOS was significantly upregulated in both tol-DC at iDC and in vitd3-tol-DC in act-DC stage. PPARγ was elevated in all DC stages with striking increase in vitd3-dexa-treated samples in mDC (Supplementary Fig. 6C).

Next, we used Rapamycin and Dorsomorphin concentrations to functionally inhibit mTOR and AMPK signaling during maturation phase in control and tolerogenic DC respectively minimal effects on cell viability (Fig. 5E, Supplementary Fig. 6D). Rapamycin inhibition of p-mTOR was confirmed during early (30 min) LPS/IFNγ activation phase (Supplementary Fig. 6E) of iDC. Rapamycin treatment reduced HLA-DR, with near significant decrease in CD86, PD-L1 in control cells and significantly reduced expression of tolerogenic marker ILT3 in vitd3-mDC samples (Fig. 5E). Dorsomorphin dramatically downregulated immune activation markers, FA transporter CD36 and significantly reduced ILT3 and CD141 in both control and vitd3-tol-DC (Fig. 5E). We also noticed that in addition to p-AMPK, Dorsomorphin also reduced total mTOR levels in both ctrl and vitd3-DC, which was previously observed in malignant cells^39^ and poses a limitation to precise interpretation of its effects in our system. Both inhibitors exhibited effects on cellular metabolism by reducing glucose consumption and lactate production. This effect appears to be more pronounced in vitd3-tol-DC as compared to inflammatory DC (Fig. 5F). These results collectively suggest that mTOR and AMPK play critical functional role and exhibit opposing regulation primarily at the actDC maturation stage. Furthermore, tolerogenic cells are more sensitive to mTOR and/or AMPK signaling inhibition.

### Blockade of lactate transport via MCT1 reduces tolerogenic phenotype of vitd3-tol-DC

Consistent with enhanced glycolytic metabolism, increased lactate production by tol-DC has been implicated in immuno-regulatory effects on T cell proliferation^40^. Since transporter MCT1 was significantly upregulated in tol-DC (Supplementary Fig. 5D) we used selective inhibitor BAY8002 to determine consequences of MCT1 inhibition on vitd3-tol-DC. BAY8002 treatment of both ctrl and vitd3-tol-DC treatment dramatically reduced lactate in culture media and affected glucose consumption by vitd3-tol-DC (Fig. 5F). While not affecting immune markers on control cells, BAY8002 significantly reduced expression of tolerogenic markers ILT3, CD141 including PD-L1 vitd3-tol-DC (Fig. 5E).

### Vitd3 and dexamethasone impacts population-specific functional metabolic states and alters dynamics of immune phenotypes

**Fig. 6:**
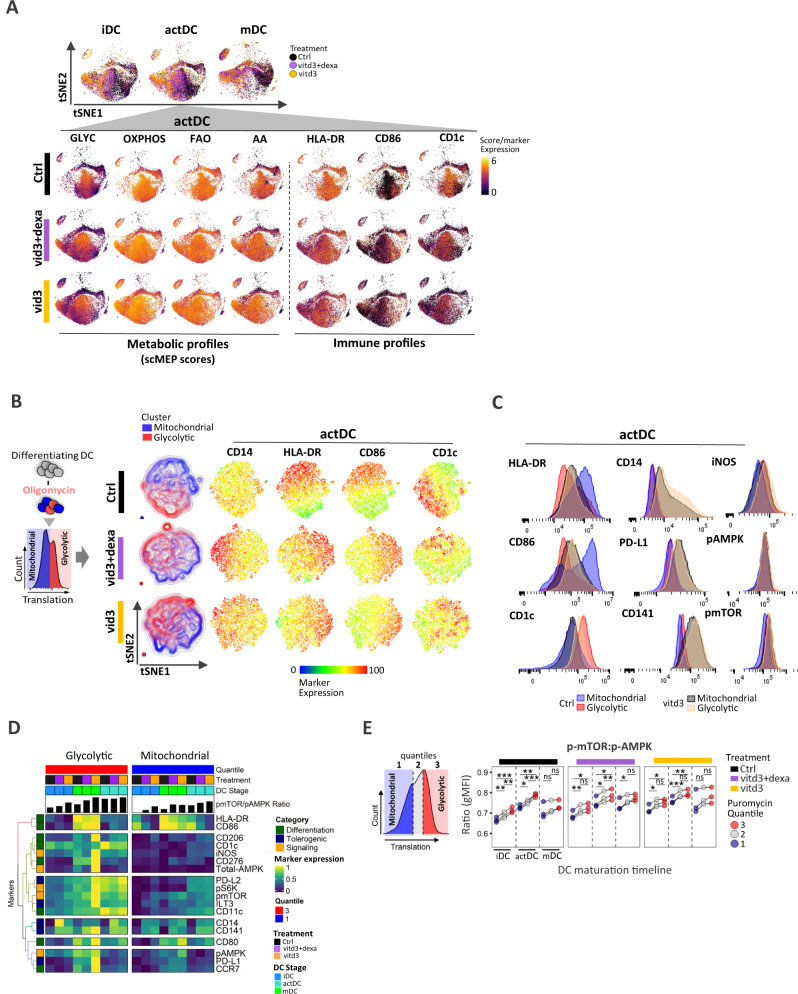
Distinct metabolic states of mitochondrial and glycolytic cell populations exhibit unique immune activation DC profiles in control and tolerogenic culture conditions. **A** tSNE maps based on metabolic marker expression of control (black), vitd3+dexa (purple) and vitd3 (orange)-treated DC across three maturation stages from one donor (out of *N* = 3 donors) are shown. Heatmap overlay of single-cell scMEP metabolic pathway scores and expression of DC-lineage markers are depicted at actDC stage to emphasize both immune and underlying metabolic heterogeneity including differences between control and tolerogenic DC. **B** Schematics of oligomycin-treated SCENITH samples, which separates cells that can effectively utilize glycolysis (red population) for producing ATP measured by protein synthesis when mitochondrial respiration is inhibited. Puromycin/ protein synthesis histograms represent cells isolated from single oligomycin-treated wells. Control (black), vitd3+dexa (purple) and vitd3 (orange)-cultured samples after oligomycin treatment exhibit glycolytic (red) and mitochondrial-dependent (blue) DC subsets in a tSNE clustering based on immune markers. Single-cell heatmap expression overlays emphasize differences in surface marker expression between glycolytic and mitochondrial DC subsets. **C** Flow-cytometry histogram profiles for differential SCENITH panel markers in glycolytic (red, orange) and mitochondrial (blue, black) populations in control and vitd3-tol-DC samples. Representative histograms from single donor (out of *N* = 3 donors) are shown. **D** Heatmap of gMFI SCENITH marker profiles in glycolytic and mitochondrial metabolic clusters from control, vitd3+dexa and vitd3 DC across distinct maturation stages. Mean expression values from three independent donors are presented. Donor label, treatment and DC differentiation stages are annotated along with the calculated mTOR:AMPK phosphorylation ratio. Marker colors represent functional categories. **E** Schematics of puromycin/protein synthesis quantile levels in oligomycin-treated SCENITH samples. Dot plots show calculated comparisons of p-mTOR:p-AMPK ratio changes between individual quantiles within respective treatment groups across maturation stages from three independent donors. Lines connect data points from an individual donor. Statistical significance was calculated via one-way ANOVA with Tukey’s *post-hoc* test. For all panels, *P*-values are represented as *p ≤ 0.05, **p ≤ 0.01, ***p ≤ 0.001, ****p ≤ 0.0001. *p*-values < 0.05 were considered statistically significant (ns). Source data are provided as a Source Data file.

To visualize population-specific metabolic heterogeneity of inflammatory vs tol-DC, we used metabolic scMEP markers to construct tSNE maps, which yielded a continuum of transitional metabolic states, depicted by a heatmap overlay of scMEP pathway scores (Fig. 6A). Spatial distribution of metabolic patterns segregated ctrl from tol-DC with reduced HLA-DR^+^, CD86^+^ and CD1c^+^. However, despite the lack of full activation of DC maturation markers in tol-DC, here we show that CD1c^hi^ and CD86^hi^ cells still retained their metabolic identity, in which CD1c^hi^ cells resided in glycolytic space, while CD86^hi^ skewed towards OXPHOS phenotype. With reduced CD1c^hi^ and CD86^hi^ cell frequency, the spatial differentials persisted at later 24h-stage of maturation (Supplementary Fig. 6F). Furthermore, expression of hallmark maturation markers HLA-DR and CD86 associated with higher mitochondrial/OXPHOS regions (Fig. 6A, B) and exhibited significant inverse relationship with glycolytic metabolism (Supplementary Fig. 7A).

Next, we analyzed individual wells from concurrently processed oligomycin-treated SCENITH samples (Supplementary Fig. 7B). As previously reported^27^ cells at the higher spectrum of translation in the presence of oligomycin are able sustain protein synthesis independent of mitochondrial respiration are cells with high glycolytic capacity (“glycolytic”; Fig. 6B). Cells which are unable to utilize or switch effectively to glycolysis are “mitochondrial-dependent” (Fig. 6B). Dimensionality reduction of oligomycin actDC using immune parameters showed polarization of mitochondrial and glycolytic cell populations in control and tol-DC (Fig. 6B).

We observed the subset of DC with the most advanced maturation phenotype, (exhibiting highest levels of HLA-DR and CD86 expression) are primarily dependent on mitochondrial respiration, while glycolytic populations were enriched for CD1c (Fig. 6C, Supplementary Fig. 7C). Importantly, cells cultured under tolerogenic conditions express only moderate levels of differentiation markers with elevated CD14, PD-L1 and CD141 irrespective of their metabolic profile. While p-AMPK was largely unaltered, both iNOS and p-mTOR levels were upregulated in glycolytic cells irrespective of treatment (Fig. 6C, Supplementary Fig. 7C). Integrative heatmap of gMFI further depicts the underlying co-expression patterns of immune and signaling markers that differentiate mitochondrial from glycolytic cell populations with respect to inflammatory or tolerogenic DC phenotype (Fig. 6D, E). Lastly, we wanted to determine the status of mTOR and AMPK phosphorylation levels in mitochondrial and glycolytic cell populations, thus we subdivided oligomycin treated data sets into 3 quantiles encompassing low, medium, and high puromycin expression as diagramed (Fig. 6E). Importantly, p-mTOR:pAMPK ratio was significantly increased in glycolytic quantile 3 DC populations at all maturation stages and treatments (Fig. 6D, E) as well as increased in tol-DC (Supplementary Fig. 7D).

### Elevated glycolytic capacity confers maturation resistance and de-differentiated immunosuppressive phenotype of tol-DC

**Fig. 7:**
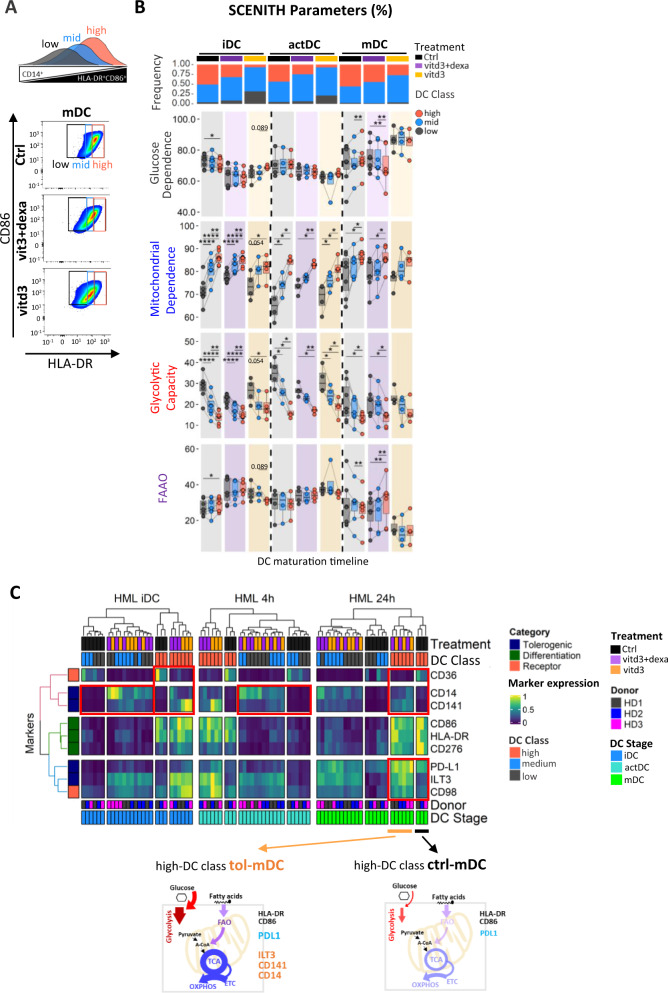
High mitochondrial dependence and low glycolytic capacity associates with increased expression of maturation markers HLA-DR^+^CD86^+^ in control but is imbalanced in tolerogenic DC. **A** Schematic depiction and gating strategy for identifying high, mid, and low HLA-DR^+^CD86^+^ expressing control, vitd3+dexa and vitd3-treated DC populations across differentiation stages. **B** Boxplots represent changes in percentual SCENITH parameters emphasizing changes between high, mid, and low HLA-DR^+^CD86^+^ populations from control (black), vitd3+dexa (purple) and vitd3 (orange)-treated DC across maturation stages (*N* = 5 donors). Lines connect data points from an individual donor. Frequencies of DC classes across individual samples are depicted at the top of the panel. Statistical significance was calculated via one-way ANOVA with Tukey’s *post-hoc* test. **C** Integrative heatmap and clustering analysis shows gMFI SCENITH immune marker profiles for high, mid, and low HLA-DR^+^CD86^+^ DC classes in control, vitd3+dexa and vitd3 samples across distinct DC maturation stages. Red boxes highlight interesting immuno-phenotypic differences between control and tol-DCs within the same DC classes. Schematics summary for metabolic and phenotypic differences between HLA-DR^+^CD86^+^ DC classes across maturation stages in control and tol-DCs. For all panels, *P*-values are represented as *p ≤ 0.05, **p ≤ 0.01, ***p ≤ 0.001, ****P ≤ 0.0001. *p*-values < 0.05 were considered statistically significant (ns). Box plots indicate second and third quantile (box), median (horizontal line) and 1.5× the interquartile range (whiskers). Source data are provided as a Source Data file.

Next, we wanted to reflect the metabolic heterogeneity associated with natural differentiation stochasticity of in vitro differentiating control DC cultures and compare these to maturation-deficiencies and deregulated metabolism of vitd3 and dexamethasone-induced tol-DC. Thus, we classified the maturation stages of both control and tol-DC into high, mid, and low quantiles based on their HLA-DR expression range (Fig. 7A, Supplementary Fig. 7E) and analyzed their SCENITH parameters. Tolerogenic conditions reduced frequencies of high-DC populations (Supplementary Fig. 7F), and we found using SCENITH that high-DC with the best differentiation quality exhibit a statistically significant increase in mitochondrial dependence and lowest glycolytic capacity irrespective of control or tolerogenic culture conditions (Fig. 7B). Clustering heatmap of immune marker expression demonstrated that tolerogenic DC do not just represent stochastically delayed DC maturation lineage (Fig. 6C). Although with diminished frequencies, tol-DC in the highest DC class are not equivalent to the inflammatory counterparts. Despite their close to equivalent levels of HLA-DR and CD86 expression, high-DC class tol-DC are marked by unique immunoregulatory receptor signatures. Furthermore, high-DC class tol-DC exhibit elevated scMEP scores particularly for OXPHOS, glycolytic and FAO pathways (Supplementary Fig. 6G). Our results suggest that metabolic reprogramming of tol-DC is not just due to a proportional switch in metabolic pathways, but rather due to overall enhancement of metabolic pathway activity.

## Discussion

Metabolism has a critical impact on DC activation, and differences in metabolic wiring have been attributed to distinct DC subtypes^14,41,42^, differentiation stimuli^43^ and T-cell priming stages (Patente et al., 2019a)^44^, murine vs human origin^11^, immunotolerance^15^, mechanical stiffness^45^ and microenvironmental influence in various pathophysiological settings^46^. Precise understanding of immunometabolic networks has been limited due to low abundance of DC subsets in the blood as well as challenges associated with bulk metabolic measurement which may not reflect natural temporal stochasticity and more recently recognized heterogeneity of ex-vivo cell cultures^22,23^. To date, multiparametric platforms have provided an excellent tool for identification of metabolic diversity in various aspects of T cell activation states and subtypes^26,29,30^. Furthermore, quantification of key metabolic proteins in the OXPHOS and glycolytic pathways predicted respective metabolic activity when combined with functional ECAR/OCR seahorse measurements^26,29,30^.

**Fig. 8:**
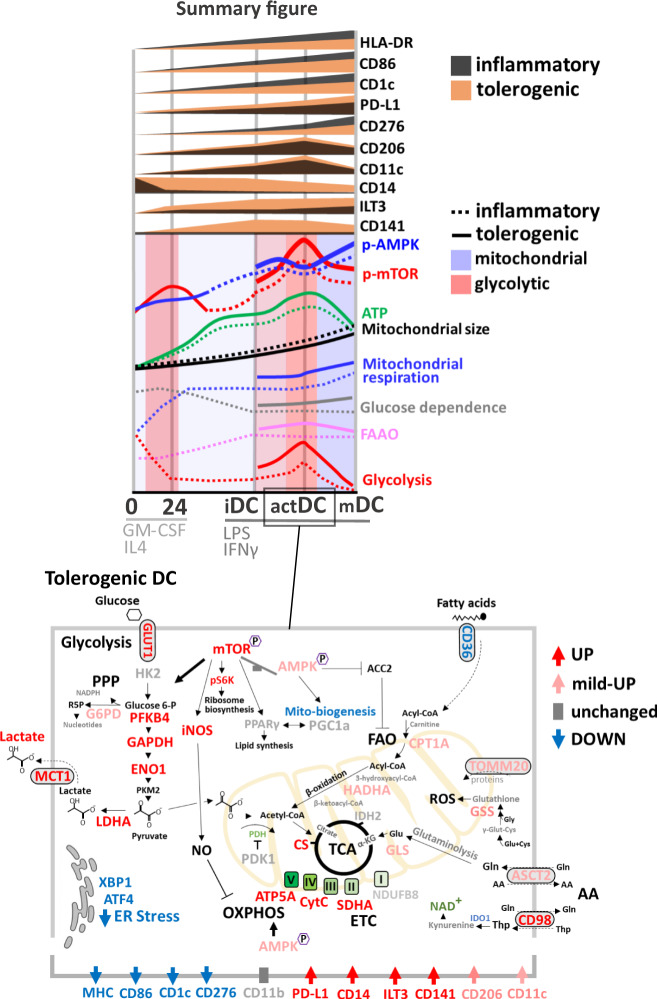
Summary diagram of immunometabolic reprogramming of inflammatory and tolerogenic DC during differentiation signaling. A schematic depiction of the metabolic and immune changes of inflammatory and tolerogenic DC is presented, noting key mechanisms and pathways.

By combining SCENITH^27^ and scMEP-based quantifying metabolic enzymes, transporters and signaling factors^29^ we show that changes in the metabolic regulome and coordinate activation of multiple metabolic pathways across distinct stages of DC differentiation and maturation are at play (Fig. 8). Upregulation of critical lineage markers associated with change from glycolytic CD14^+^ precursors to mitochondrial metabolism during initial 24 h of GM-CSF/IL4 differentiation, which persisted across DC stages reaching 86% in mDC. Contrary to TLR-induced activation of murine BMDCs, characterized by a maximal upregulation of glycolysis with repressed OXPHOS and collapsed mitochondrial activity^5,8^, maturation of human monocytic DC undergo only moderate and transient increase in glycolytic metabolism, which similarly observed in a study by Malinarich et al.^12^. Fully matured DC had lowest glycolytic capacity (14%) and exhibited increased glutaminolysis dependence (41%) compared to FA oxidation (7%).

Analysis of SCENITH metabolic profiles were complemented by coordinate activation of multiple measured components of the TCA/ETC pathway, FAO markers CPT1A, HADHA together with AA transporters ASCT2, CD98 and glutaminolysis enzyme GLS. In support of active mitochondrial biogenesis and elevated respiratory complex-dependent ROS formation during DC differentiation^47,48^, we observed elevated expression of PGC1α, TOMM20 and antioxidant GSS persisting through maturation.

To establish relationships between enzyme and metabolite transporter expression and metabolic pathway dependence we benchmarked co-expression patterns of selected scMEP markers with protein synthesis-adjusted SCENITH parameters. The highest correlative metabolic analytes were used to define scMEP metabolic scores. While quantification of metabolic proteins robustly indicated cellular capacity for respective metabolic pathways in our study and multi-dimensional analyses of whole blood PBMC and T cells^26,29^, we noted that not all measured regulators correlated with SCENITH functional measurements. Out of 5 measured ETC/TCA regulators, CytC was the least correlative with mitochondrial dependence, and beta-oxidation pathway enzyme HADHA and AA metabolism components ASCT2, CD98 and GLS were the highest correlating enzymes with FAAO capacity. We observed dichotomous pattern of glycolytic enzymes with both, constitutive and inducible expression profiles out of which, MCT1 and PFKFB4 were highest correlating enzymes with glycolytic capacity across DC differentiation.

Monocytes maintained their glycolytic metabolism ex-vivo^49^ and exhibited high expression of ENO1, GAPDH and LDHA, which decreased during DC differentiation. In contrast, glycolytic scMEP markers regulating glucose import (GLUT1), phosphorylation (PFKFB4) and lactate export (MCT1) increased with DC maturation and have been previously defined as primary drivers of flux through glycolysis^50^.

While our scMEP analysis provides insights into the metabolic regulome of inflammatory and tolerogenic DC differentiation, our scMEP panel contained only a subset of metabolic regulators. The discrepancy between expression of certain scMEP factors and SCENITH functional measurements suggests that additional isoenzymes, metabolite transporters and/or post-translational modifications are at play and should be further evaluated in future studies.

An important factor to consider is that metabolism of in vitro cultured cells may not properly reflect cellular metabolism in vivo, given the high concentration of glucose in media. Our comparative SCENITH analysis revealed in vitro cultured inflammatory DC exhibited highest glucose dependence (90%) as compared to total DC populations, pDC and cDC1 from freshly isolated PBMCs. While these differences may be due to inherent differences in metabolic reprograming of specific DC subsets^14^, we did observe that DC populations from PBMCs exhibited higher FAAO capacity (65%), suggesting their heightened utilization of FAO and Glutaminolysis as an energy source in vivo. Our results are consistent with a study by Patente et al.^51^, demonstrating that TLR-stimulation causes increased mitochondrial content with high OXPHOS fueled by glutamine metabolism in pDC.

Single-cell metabolic score profiling enabled us to monitor dynamics of multiple pathways in cell populations across differentiation timeline. In contrast to homogenous co-activation of OXPHOS, AA, FAO, mitochondrial dynamics and glutathione synthesis pathways following LPS/IFNγ activation glycolysis scMEP scores spanned a wider range of metabolic heterogeneity. This was directly related to divergent immune phenotypes of maturing DC with CD86^Hi^ and CD1c^Hi^ populations exhibiting preferential enrichment toward OXPHOS and glycolytic metabolism, respectively. Phenotypically, CD86^hi^ DC exhibited higher expression of DC markers HLA-DR, CD206, PD-L1 and CD276. Our results demonstrating that glycolytic metabolisms underlies polarization of CD1c expression in ex vivo differentiating DC is consistent with a recent study by Basit et al.^14^, demonstrating that TLR7/8-stimulation of circulating CD1c^+^ DC induced mitophagy-dependent shift towards glycolysis with reduced expression of OXPHOS-related genes and mitochondrial content. We further show that elevated glycolytic capacity in CD1c^hi^ DC is associated with increased abundance of GLUT1, MCT1 and PFKFB4, and elevated PDK1, which skews glucose homeostasis^35^ by preventing pyruvate shuttling into TCA into mitochondria.

Regulation of activation states of p-AMPK in the context of human inflammatory DC is largely unknown and its associations with immature and tolerogenic DC phenotype are primarily based on *AMPK1α* mRNA^12,20^ and the use of pharmacological activator AICAR did not yield conclusive results^20^, which may partly by due to its AMPK-independent effects including blockade of NF-κB transactivation^52,53^. AMPK activation was shown to antagonize mTORC1 signaling and glycolytic switch in murine BMDC^5^ and its inactivation fostered inflammatory function and maturation of murine macrophages and myeloid APC^54^. Kratchmarov et al.^55^, showed that APMK modulated Flt3L-induced progenitor development into cDC1/cDC2 cell fate and AMPK/TRV activation was reported to mediate the suppressive effects of oleoylethanolamide on TLR4/NF-κB-dependent BDMC maturation^56^. In a recent study, CCR7-engagement blocked the pro-apoptotic role of AMPK and promoted survival of mDC^57^. Contrary to the anti-inflammatory role in murine DC, activation of AMPK was recently shown to support OXPHOS reprogramming and interferon type I & III production in TLR7/9 activated human pDC^58^. Therefore, we profiled kinetic phosphorylation changes for mTOR and AMPK across DC differentiation. AMPK phosphorylation which correlated with increased mitochondrial dependence in mDC opposed p-mTOR levels, which paralleled LPS/IFNγ-induced transient increase in glycolytic capacity and FAAO in actDC. The opposing phosphorylation patterns were reflected in significant changes in p-mTOR:p-AMPK ratio across maturation stages. Inhibition of mTOR and AMPK signaling at the time of LPS/IFNγ-induced maturation reduced inhibited lactate production and prevented upregulation of critical immune surface markers HLA-DR, CD86 and PD-L1 on DC. While recent studies now demonstrate possible concurrent activation of AMPK and mTOR by amino acids^59^, we propose that time-specific regulation of mTOR and AMPK phosphorylation underlies metabolic reprogramming across distinct differentiation stages of human DC.

Tolerogenic DC have been evaluated as cellular products for treatment of multiple autoimmune diseases^25^. However, reports using a variety of protocols used to generate tol-DC in vitro^60^ indicated that metabolic plasticity and the heterogeneous nature associated with inherent epigenetic and transcriptional reprogramming is a cofounding factor in precise understanding of tol-DC^38,61^. Along with increased glucose consumption and lactate production, our SCENITH analysis confirmed persistent in increase glycolytic capacity, glucose dependence as well as transient increase in mitochondrial dependence and FAAO capacity in tol-DC. Elevated glucose consumption was shown to fuel glycolysis and TCA cycle^25,62,63^ and maintain tolerogenic phenotype of vitd3-tol-DC^20^. scMEP analysis further revealed simultaneous upregulation of TCA/ETC, glycolysis and AA metabolic scores particularly in the vitd3-tol-DC. This recapitulated previous studies^12,37,62,63^ and further showed that specific metabolic pathways are already elevated at the iDC stage and transient in nature following tol-DC maturation, which was not previously described. In fact, tol-iDC and 4h-activated tol-mDC exhibited the highest diversity in metabolic pathway markers including upregulation of FAO (CPT1α, HADHA), mitochondrial dynamics and components of glutamine metabolism regulating its transport (ASCT2) and conversion to TCA cycle intermediate α-ketoglutarate (GLS)^64^.

Importantly, we demonstrate that persistent increase in mTOR:AMPK phosphorylation ratio activation reflects metabolic hyperactivation of tol-DC which is consistent with critical role for PI3K/Akt/mTOR pathway in promoting vitd3-induced glycolytic reprogramming and tolerogenic effects on DC^20^. Inhibition of mTOR and AMPK using Rapamycin and Dorsomorphin respectively, significantly decreased tolerogenic marker expression in vitd3-mDC samples. While the use of Dorsomorphin is more challenging to interpret because it also reduced total mTOR levels, targeting mTOR signaling significantly and to greater extend reduced glucose consumption and lactate production in vitd3-tol-DC as compared to inflammatory DC. Because lactate exerts immune-suppressive effects on T cells^40^, we asked whether blockade of lactate transporter MCT1 induces changes in metabolic and tolerogenic immune phenotype of DC. Blockade of MCT1 dramatically reduced lactate levels with modest effects on glucose consumption by DC. While not affecting immune markers on inflammatory DC, MCT1 inhibition significantly reduced expression of tolerogenic markers ILT3, CD141 including PD-L1 vitd3-tol-DC. This data provide evidence that modulations of cellular metabolism by targeting AMPK:mTOR signaling axis and/or inhibiting lactate transport influence tolerogenic phenotype of DC.

In this study, high-dimensional techniques enabled us to simultaneous profiling of metabolic and immune phenotypes in inflammatory and tol-DC development at the single-cell level. Using oligomycin-treated single-cell experiments, we demonstrated that glycolytic metabolism underlies transitional and less-well matured immune phenotypes expressing moderate HLA-DR and CD86, which resembled maturation-deficient tol-DC levels. Because we observed that a wide range of OXPHOS and glycolytic scores represent metabolic heterogeneity of single culturing wells, we separated low, mid and high HLA-DR and CD86 expressing cells and compared their metabolic and immune features between inflammatory and tolerogenic conditions. We examined whether maturation delays and stochastic heterogeneity in inflammatory DC parallels “maturation resistant” immune phenotype of tol-DC. SCENITH profiling HLA-DR and CD86 high cells in both control and tolerogenic cultures exhibited similar metabolic pathway percent activation with highest OXPHOS and lowest glycolytic capacity. scMEP analysis further revealed that augmented OXPHOS, glycolysis and FAO activation is at play in vitd3 and dexa-vitd3-tol-DC. In addition to augmented metabolism, we revealed that tol-DC with the highest differentiation quality are marked by unique immunoregulatory receptor signatures, which do not reflect maturation delays of in vitro inflammatory DC. Therefore, we propose that tol-DC are not only locked in a “maturation-resistant” state with reduced expression of DC-lineage markers, but also resemble a cross-differentiated phenotype by retention of CD14 and increased CD141 and immunosuppressive checkpoint receptors PD-L1 and ILT3^65,66^.

The use of single-cell high-dimensional techniques enabled us to validate as well as capture previously obscured immune-metabolic diversity of DC. Our results provide a basis for future monitoring of the metabolic states underlying phenotypic heterogeneity of immunogenic and tolerogenic DC physiology for management and improvement of DC-based immunotherapies^51^.

## Methods

### In vitro monocytic DC generation

PBMCs from healthy donors were purchased (Trima Residuals RE202, Vitalant) and purified by Ficoll-hypaque gradient centrifugation (Fisher Scientific, 45-001-749). Cryopreserved PBMCs were thawed using RPMI (Gibco-Invitrogen) complete media (1% Pen Strep, 1% L-Glutamine, 10% FBS Heat Inactivated Serum (Gibco-Invitrogen, 16000-044), and 0.5% DNase (Sigma, DN-25) and washed twice with PBS. CD14^+^ monocytes were selected using CD14 microbeads (Miltenyi Biotec, 130-050-201) and cultured for 5 days in CellGenix medium (0020801-0500) supplemented with 800 U/mL GM-CSF (Miltenyi Biotec, 130-095-372) and 500 U/mL IL4 (Miltenyi Biotec, 130-095-373) to generate iDC). At day 3, half of media was replaced and supplemented with fresh cytokines. iDC were matured on day 5 with 1000 U/mL IFN-γ (Peprotech, 300-02) and 250 ng/mL LPS (Sigma-Aldrich, L2630). Two types of tol-DC were generated. To obtain vitd3-tol-DC 100 nM of vitamin D_3_ (Sigma, D1530) was added to cultures at d0 and day 3. And dexa-vitd3-tol-DC were generated by adding 100 nM of vitamin D_3_ and 10 nM of dexamethasone (Sigma, D4902) at day 3 to cultures. Both tol-DC were matured as described above. Rapamycin (Selleckchem, S1039), Compound C (Selleckchem,7306) and BAY8002 (Selleckchem, S 8747) were added at iDC stage together with IFN-γ/LPS for 24 h.

### SCENITH cell staining and data acquisition

SCENITH was performed as described in^27^. SCENITH™ reagents kit (inhibitors, puromycin and antibodies) were obtained from www.scenith.com/try-it and used according to the provided protocol for in-vitro derived myeloid cells. Briefly, 1 × 10^6^ PBMC or monocytic DC cultures (2 × 10^5^/24-well plate) harvested at desired time points, were treated for 18 min with Control (DMSO), 2-Deoxy-Glucose (2-DG; 100 mM), Oligomycin (O; 1 µM), Etomoxir (4 µM) (Selleckchem, S8244), CB-839 (3 µM) (Selleckchem, S7655), a combination of 2DG and Oligomycin (DGO) or Harringtonine (H; 2 µg/mL). Following metabolic inhibitors, Puromycin (final concentration 10 µg/mL) was added to cultures for 17 min. After puromycin treatment, cells were detached from wells using TypLE Select (Fisher Scientific, 505914419), washed in cold PBS and stained with a combination of Human TureStain FcX (Biolegend, 422301) and fluorescent cell viability dye (Biolegend, 423105) for 10 min 4 °C in PBS. Following PBS wash step, primary antibodies against surface markers were incubated for 25 min at 4 °C in Brilliant Stain Buffer (BD Biosciences, 563794). Cells were fixed and permeabilized using True-Nuclear Transcription Factor Buffer Set (Biolegend, 424401) as per manufacturer instructions. Intracellular staining of puromycin and protein targets was performed for 1 h in diluted (10×) permeabilization buffer at 4 °C. Finally, data acquisition was performed using the Cytek Aurora flow cytometer. Primary conjugated antibody information used in SCENITH panel is listed in supplementary table 1. All antibodies were titrated to reduce spillover and increase resolution using single stained DC (generated as described above) samples. Unstained cell controls used for autofluorescence extraction were generated for each time point, culture conditions (control, vitd3-tol-DC and dexa-vitd3-tol-DC) and metabolic inhibitor treatments (C, 2DG, O, DGO). Samples were unmixed using reference controls generated in combination with stained Ultracomp beads (Fisher Scientific, 01-2222-41) and stained cells using the SpectroFlo Software v2.2.0.1. The unmixed FCS files were used for data processing and analysis using FlowJo (BD, version 10.8.1) and CellEngine (CellCarta). Manually gated CD14^-^HLA-DR^+^CD86^+^ cells were used for downstream analysis. gMFI expression values were imported into R environment for correlation and heatmap clustering analyses using the below described R packages. Calculations used to derive SCENITH parameters:

1. [C = gMFI of anti-Puro-Fluorochrome upon Control treatment]
2. [2DG = gMFI of anti-Puro-Fluorochrome upon 2DG treatment]
3. [O = gMFI of anti-Puro-Fluorochrome upon Oligomycin treatment]
4. [Eto = gMFI of anti-Puro-Fluorochrome upon Etomoxir treatment]
5. [Tele = gMFI of anti-Puro-Fluorochrome upon CB-839 treatment]
6. [DGO = gMFI of anti-Puro-Fluorochrome upon 2DG + Oligomycin (DGO) treatment]
7. [Glucose dependence = 100(C – 2DG)/(C-DGO)]
8. [Mitochondrial dependence = 100(C – O)/(C-DGO)]
9. [FAO dependence = 100(C − Eto)/(C-DGO)]
10. [Glutaminolysis dependence = 100(C – Tele)/(C-DGO)]
11. [Glycolytic Capacity = 100 − Mitochondrial dependence]
12. [FAAO = 100 − Glucose dependence]

### Single-cell metabolic regulome profiling (scMEP) by mass cytometry

scMEP analysis was performed as recently described^29^. In short, monocytes and DC cultures were plated (2.5 × 10^6^/6-well plate) and harvested at desired time points. Antibodies targeting metabolic features were conjugated in-house using an optimized conjugation protocol^67^ and validated on multiple sample types. Cells were prepared for scMEP analysis by incubation with small molecules to be able to assess biosynthesis rates of DNA, RNA and protein, cisplatin-based live/dead staining, PFA-based cell fixation and cryopreservation (dx.doi.org/10.17504/protocols.io.bkwkkxcw). Next, cells were stained with metabolic antibodies in a procedure that includes surface staining for 30 min at RT, PFA-fixation for 10 min at RT, MeOH-based permeabilization for 10 min on ice, intracellular staining for 1 h at RT and DNA intercalation (dx.doi.org/10.17504/protocols.io.bntnmeme). Finally, cells were acquired on a CyTOF2 mass cytometer (Fluidigm). Protein targets and antibody information used in scMEP are listed in supplementary table 2.

### Mass cytometry data processing and analysis

Raw mass spectrometry data were pre-processed, de-barcoded and imported into R environment using the flowCore package (version 2.0.1)^68^. Values were arcsinh transformed (cofactor 5) and normalized^29^ for downstream analyses based on previously reported workflow^69^. Mean cell radius (forward scatter from Cytek analysis, FSC-A) was used to calculate changes in cell volume across DC differentiation. Expression of scMEP factors was normalized to account for increase in cell volume from precursors to mature DC. All clustering analyses were performed on subsampled (20–25,000 cells/treatment time point) HLA-DR^+^CD86^+^-gated cell populations with indicated input markers. Multi-dimensional plots were generated using R package limma (version 3.44.) and dimensionality reduction analysis was performed using Rtsne (version 0.15) Uniform Manifold Approximation and Projection (UMAP) was performed using FlowJo. For visualization and heatmap clustering we utilized R packages ggplot2 (version 3.3.3) and ComplexHeatmap (version 2.4.3)^70^, respectively. Differential expression analysis of marker expression between treatment groups was determined separately for each DC maturation time point using linear mixed effect model accounting for donor variability using the lme4 (version 1.1–26) package. Spearman correlation coefficient correlation matrix for marker expression profiles was computed and visualized using the corrr (version 0.4.3), Hmisc (version 4.5.0) and corrplot (version 0.88) R packages.

### Calculation of scMEP pathway scores

To determine OXPHOS, glycolysis, FAO and AA scMEP pathway scores we applied linear regression analysis between the scMEP median metabolic marker expression (arcsinh transformed) and log-transformed median SCENITH parameters (adjusted to protein synthesis) using the R package lmerTest (version 3.13). For calculating scMEP scores, the most significant and positively correlated markers within each metabolic pathway were summarized and divided by the number of markers within that pathway. Spearman correlations between scMEP pathway scores and SCENITH parameters were represented using the ggpubr (version, 0.4.0) R package.

scMEP markers used to derive respective scores include: TCA/ETC score (CS, ATP5A, IDH2), FAO score (HADHA), amino acid score (ASCT2, CD98, GLS), glycolysis predictive score (MCT1, PFKFB4), glycolysis upregulated score (GLUT1, MCT1, PFKFB4), GLYC-CON constitutive score (ENO1, LDHA, GAPDH), glutathione (GSH) biosynthesis score (GSS), mitochondrial dynamics score (TOMM20, PGC1a), pentose phosphate pathway sore (G6PD).

### Mean gini score calculations

Random forests were trained individual cells from on CyTOF dataset using the package randomForest (version, 4.6–14) by randomly selecting 80% of the cells in each sample then comparing ctrl vs vid3+dexa, ctrl vs vid3 and vid3 vs vid3+dexa treatments, at three time points (iDC, actDC and mDC) in triplicate at different starting seed values for a total of 27 unique models^71^. The Gini indices were determined for each model and a mean of the triplicate was as a representative as a “Mean Gini Score”.

### Mitochondrial mass analysis

To evaluate mitochondrial mass, live control and tol-DC (2 × 10^5^/24-well plate), cultured as described above, were stained using MitoTracker™ Deep Red FM (Invitrogen, M22426) together with surface antibody staining for 20 min at 4 C. Mitochondrial staining was measured in the APC channel, and MFI was used to obtain mitochondrial content.

### Extracellular glucose and lactate measurements

Glucose and lactate levels were analyzed in DC culture supernatants using the BG1000 Blood Glucose Meter & test strips (Clarity, 75840–796) and Blood Lactate Measuring Meter Version 2 test strips (Nova Biomedical, Lactate Plus), respectively.

### Statistical data analysis

Multiple group statistical comparisons were analyzed using one-way ANOVA with Tukey’s *post-hoc* test. *P*-values are represented as *p ≤ 0.05, **p ≤ 0.01, ***p ≤ 0.001, ****p ≤ 0.0001. *p*-values < 0.05 were considered statistically significant. Numerical labels indicate near significant values. Box plots indicate second and third quantile (box), median (horizontal line) and 1.5× the interquartile range (whiskers).

Differential SCENITH/scMEP marker expression between treatment groups within each DC maturation stage, was analyzed using the linear mixed effect model with fixed effects for treatment, and random intercepts per donor. Volcano plots depict the magnitude and adjusted *p*-values of the treatment effect. Points above the solid gray horizontal line indicate markers that are significant at the *p* < 0.05 level after multiple-testing adjustment. R (version 4.0.2) was used for statistical testing utilizing package rstatix (version 0.7.0) and graphs and significance labels were generated using ggplot2 (version 3.3.3) and ggpubr (0.4.0) respectively.

### Reporting summary

Further information on research design is available in the Nature Research Reporting Summary linked to this article.

## Supplementary information

Supplementary Information

Reporting Summary

## Data Availability

The data generated in this study are provided in the Supplementary Source Data files and available from the corresponding authors upon request. Source data are provided with this paper.

## Source data

Source Data

## Code availability

Code, data files and workflows generated for these analyses are available in a GitHub repository at https://github.com/ButterfieldLab/Nat.Comm.TolDC.project. Source data for scMEP CyTOF analysis are provided in within the source data file.
