# Supplementary material for: Distinct metabolic states guide maturation of inflammatory and tolerogenic dendritic cells
Source: Nat Commun. 2022 Sep 2;13:5184. doi: 10.1038/s41467-022-32849-1 (PMC9440236; doi:10.1038/s41467-022-32849-1)
Supplement: Supplementary file 1 — Supplementary Information [file 41467_2022_32849_MOESM1_ESM.pdf]

Supplementary Figure 1

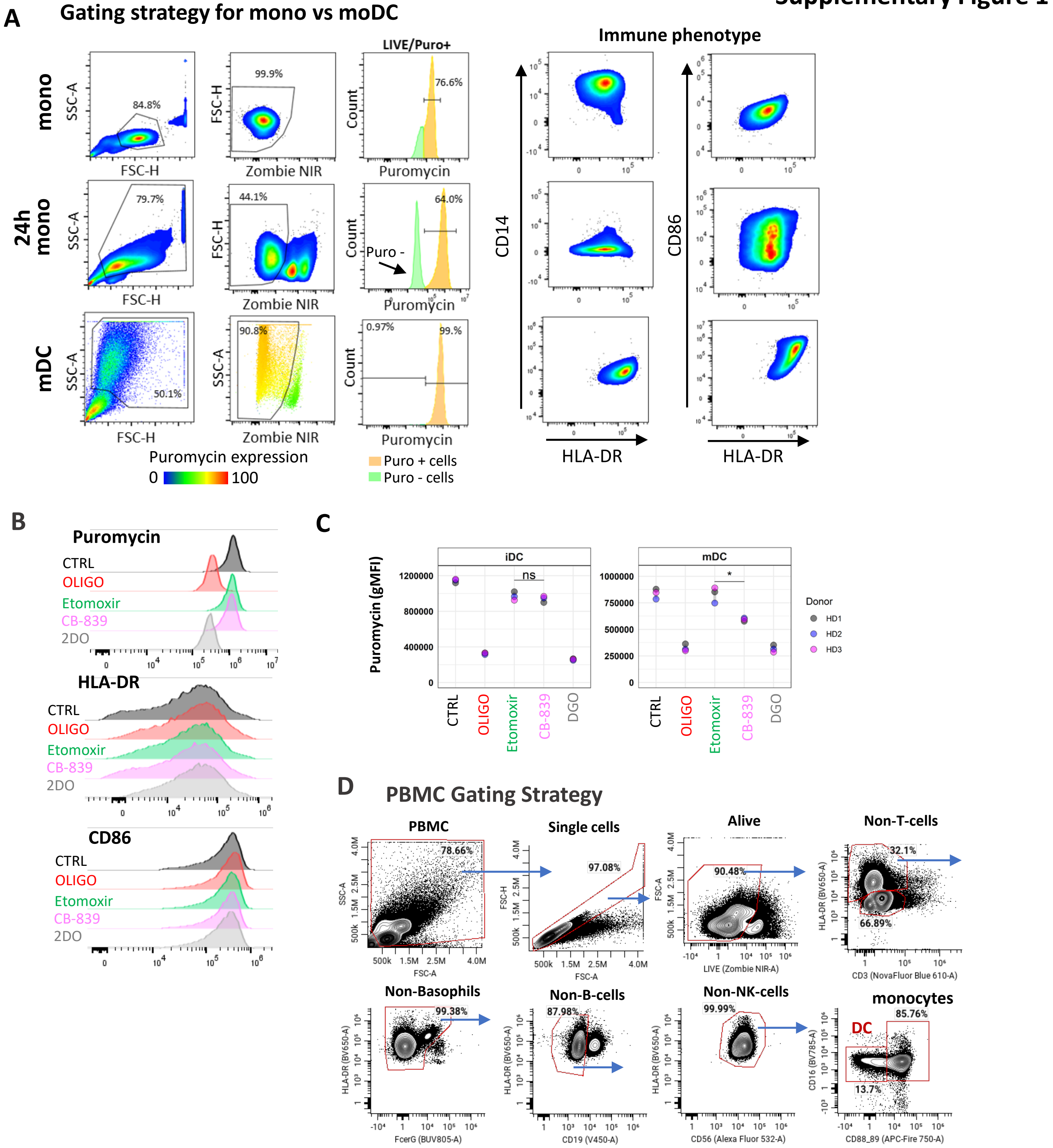

Supplementary Figure 1. Gating strategies and metabolic inhibitor effects on puromycin expression. **(A)** Gating strategies used to determine CD14^{+} HLA-DR^{-}CD86^{-} monocyte (top), 24h post-GM-CSF/IL4 stimulus CD14^{+} HLA-DR^{Lo} CD86^{Lo} precursor (middle) and mDC CD14^{-}HLA-DR^{+}CD86^{+} populations (bottom). Puromycin^{+} populations (yellow peaks) were selected for downstream analyses. **(B)** Flow cytometry histograms for Puromycin, HLA-DR and CD86 expression changes in control DC treated with indicated metabolic inhibitors. **(C)** Bar graphs with mean±SE represent gMFI of Puromycin expression changes in control and metabolic inhibitor samples (bar graphs represent 3 independent replicates from 1 donor). Statistical significance was calculated using two-sided Student's t-test. **(D)** Gating strategies for immune characterization of freshly isolated blood monocytes and DC populations. Representative contour plots for 1 donor (out of N = 3) are shown.

Supplementary Figure 2

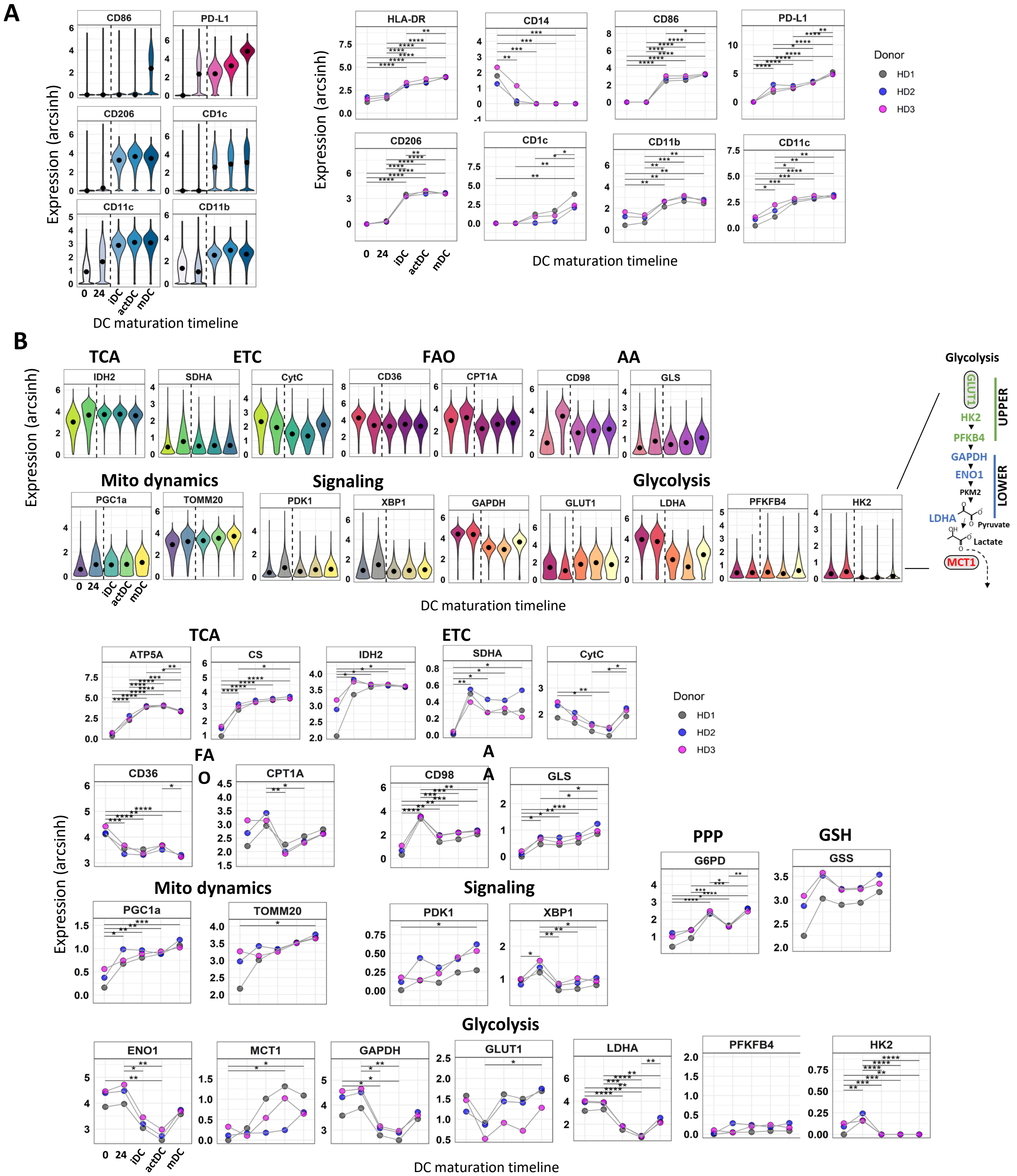

Supplementary Figure 2. Expression of scMEP metabolic markers and single cell scores across differentiation DC timeline. Shown are (arcsinh transformed) expression values for **(A)** scMEP immune markers and **(B)** scMEP metabolic markers across DC differentiation states. Black dots represent population medians and the dotted line separates early precursors from iDC, actDC, and mDC stages. Violin plots are representative of 1 donor (out of N = 3). Dot plots on right represent median scMEP marker expression values for 3 donors with denoted statistical significance, calculated using one-way ANOVA with Tukey's *post-hoc* test.

Supplementary Figure 3

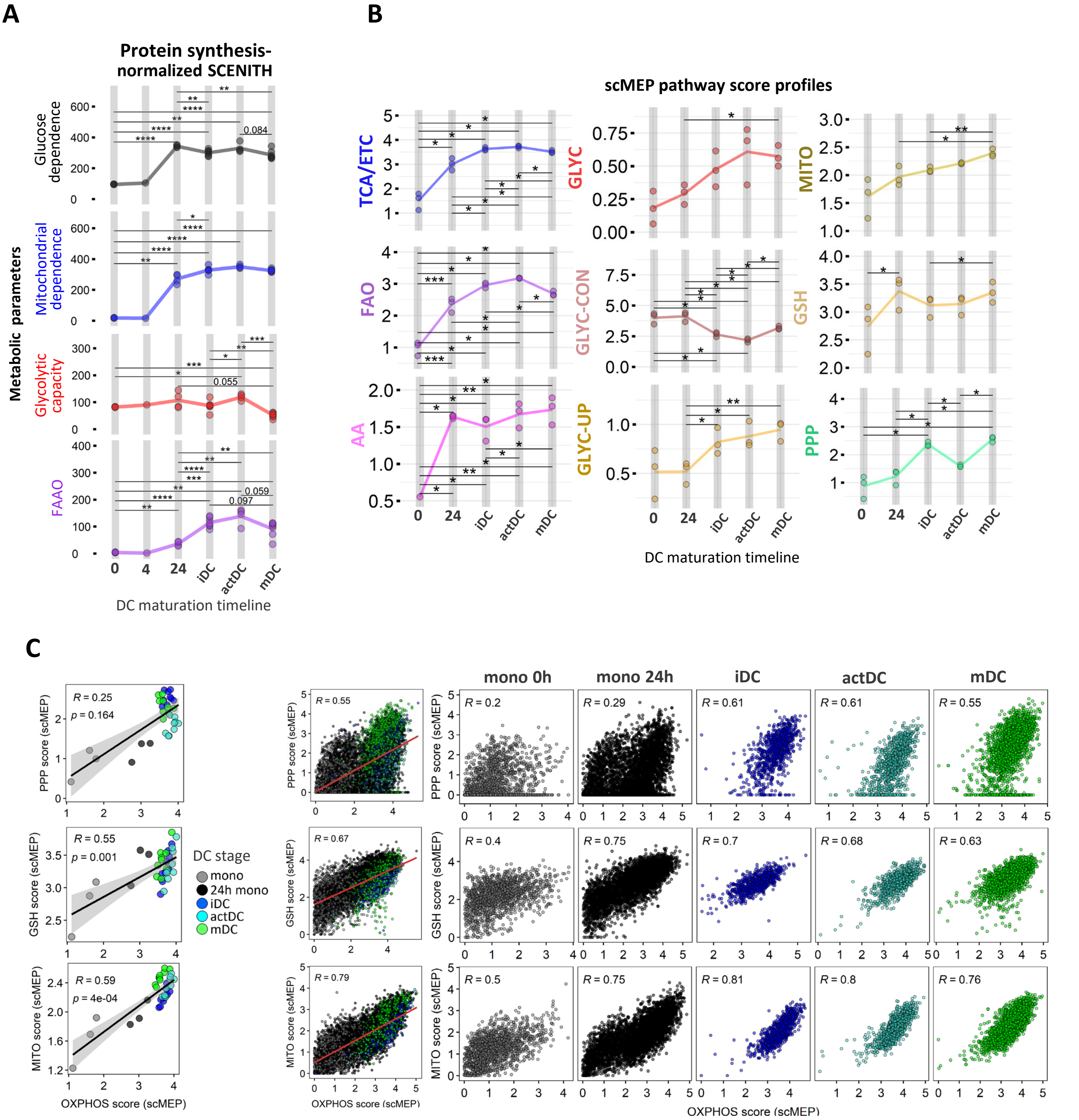

Supplementary Figure 3 Associations between single-cell scMEP metabolic pathway scores and immune phenotypes of DC differentiation stages. **(A)** Overview of kinetic changes in protein synthesis-adjusted percentual SCENITH parameters and **(B)** scMEP metabolic scores across DC differentiation timeline with indicated significance for pairwise statistical comparisons. Lines represent average SCENITH profiles (precursor stages represent 3 independent donors, iDC, actDC, and mDC represent 6 independent donors). scMEP samples are representative of 3 individual donors. **(A, B)** Statistical Significance was calculated using one-way ANOVA with Tukey's *post-hoc* test. **(C)** Correlations between median SCENITH parameters and respective calculated median scMEP pathway scores with Spearman correlation coefficient (R), p-value and grey shading denoting 95% confidence interval (CI). Middle and multi-panel graphs depict single-cell scMEP scores for combined and individual DC sample time points respectively. Subsampled single-cell data points for the individual donor (out of N = 3) are shown.

Supplementary Figure 4

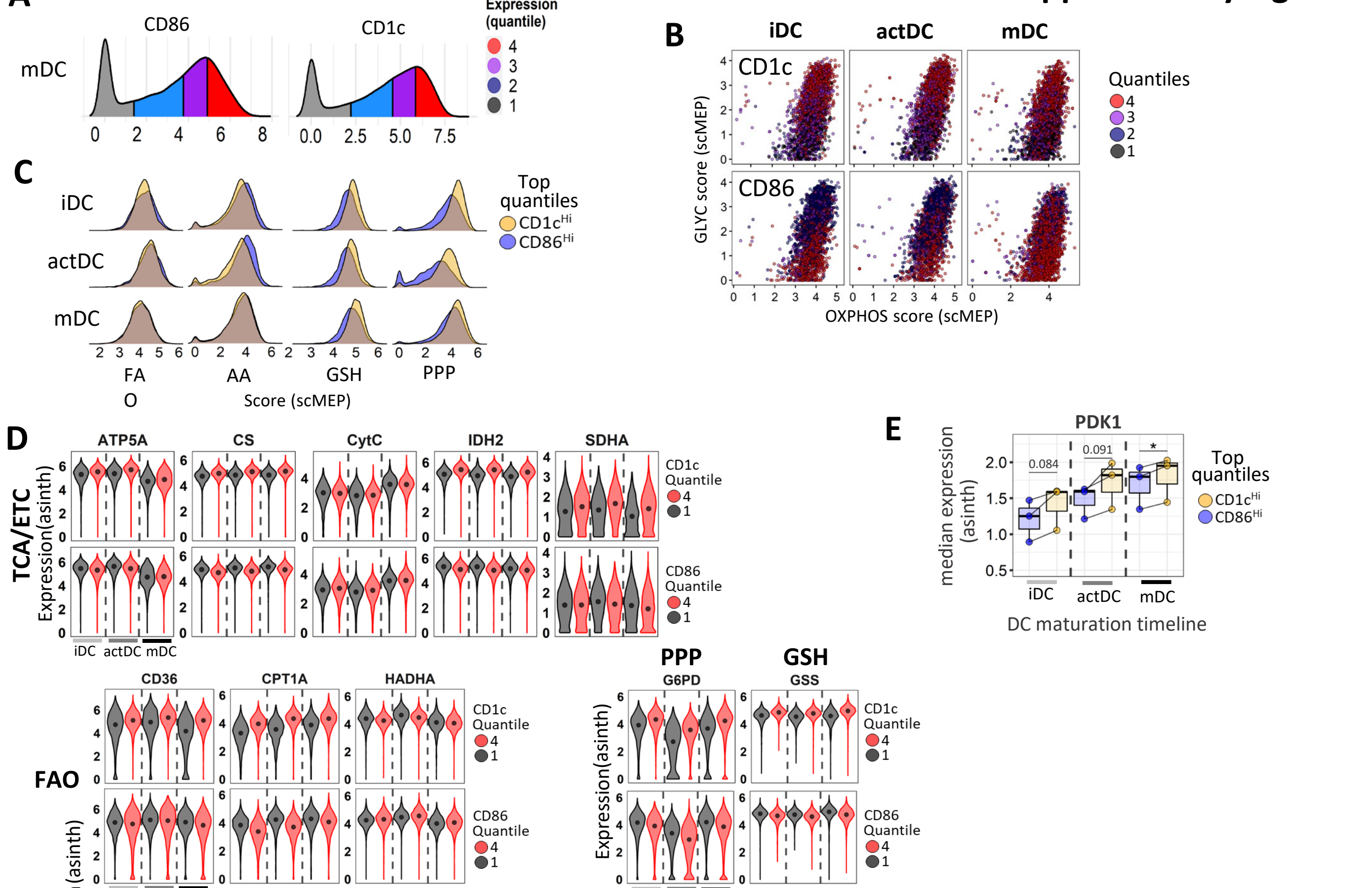

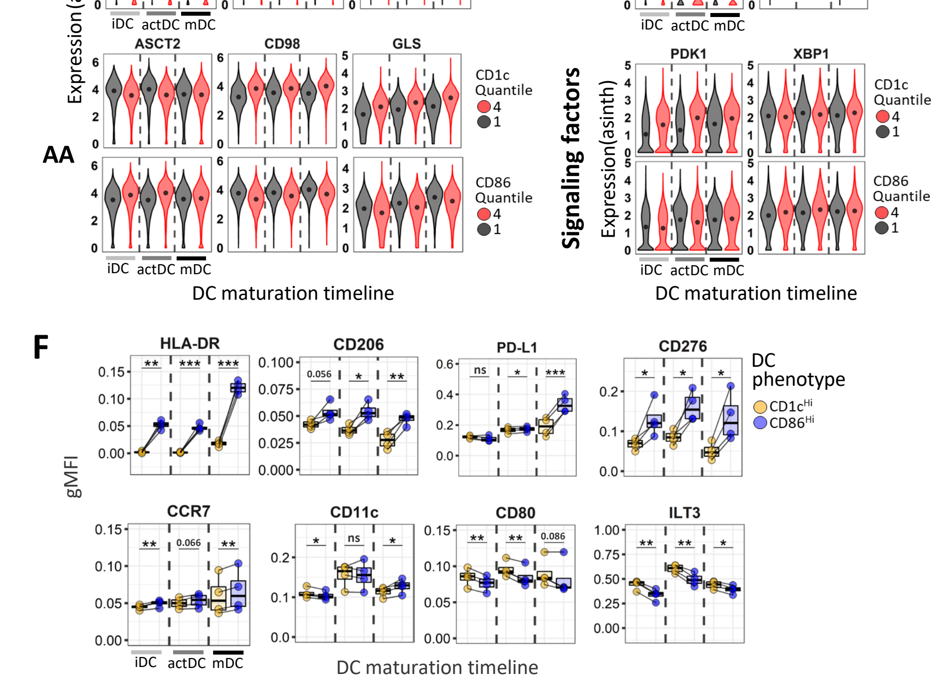

Supplementary Figure 4 Immuno-metabolic phenotypes of CD1c^{hi} and CD86^{hi} DC populations across DC differentiation stages. **(A)** Histograms depicting quantile ranges of arcsinh transformed CD86 and CD1c expression profiles in mDC used for downstream subsampling and exploratory analyses. **(B)** Single-cell values for individual CD1c and CD86 markers were divided into four quantiles spanning the lowest (black circles) and highest (red circles) expression levels. CD1c and CD86 quantiles were overlayed on single-cell scMEP GLYC vs OXPHOS correlation scatterplots to emphasize dichotomous stratification of CD1c^{hi} and CD86^{hi} DC populations.

(C) Shown are histogram distributions of single-cell scMEP metabolic pathway scores in CD1c^{hi} (blue) and CD86^{hi} (gold) DC populations. **(D)** Expression values of scMEP-profiled enzymes/metabolite transporters and signaling factors from indicated metabolic pathways in the 1st (lowest, black) and 4th (highest, red) quantile from CD1c and CD86 populations across iDC, actDC, and mDC. Figures **(A-D)** represent single cell data points from one donor (out of N = 3). **(E)** Shown are median expression values for PDK1 and **(F)** immune markers in the top 4th quantiles from CD1c^{hi} (blue) and CD86^{hi} (gold) DC populations across differentiation stages. **(E-F)** represent data points from 3 independent donors with statistical Significance calculated using one-way ANOVA with Tukey's *post-hoc* test.

Supplementary Figure 5

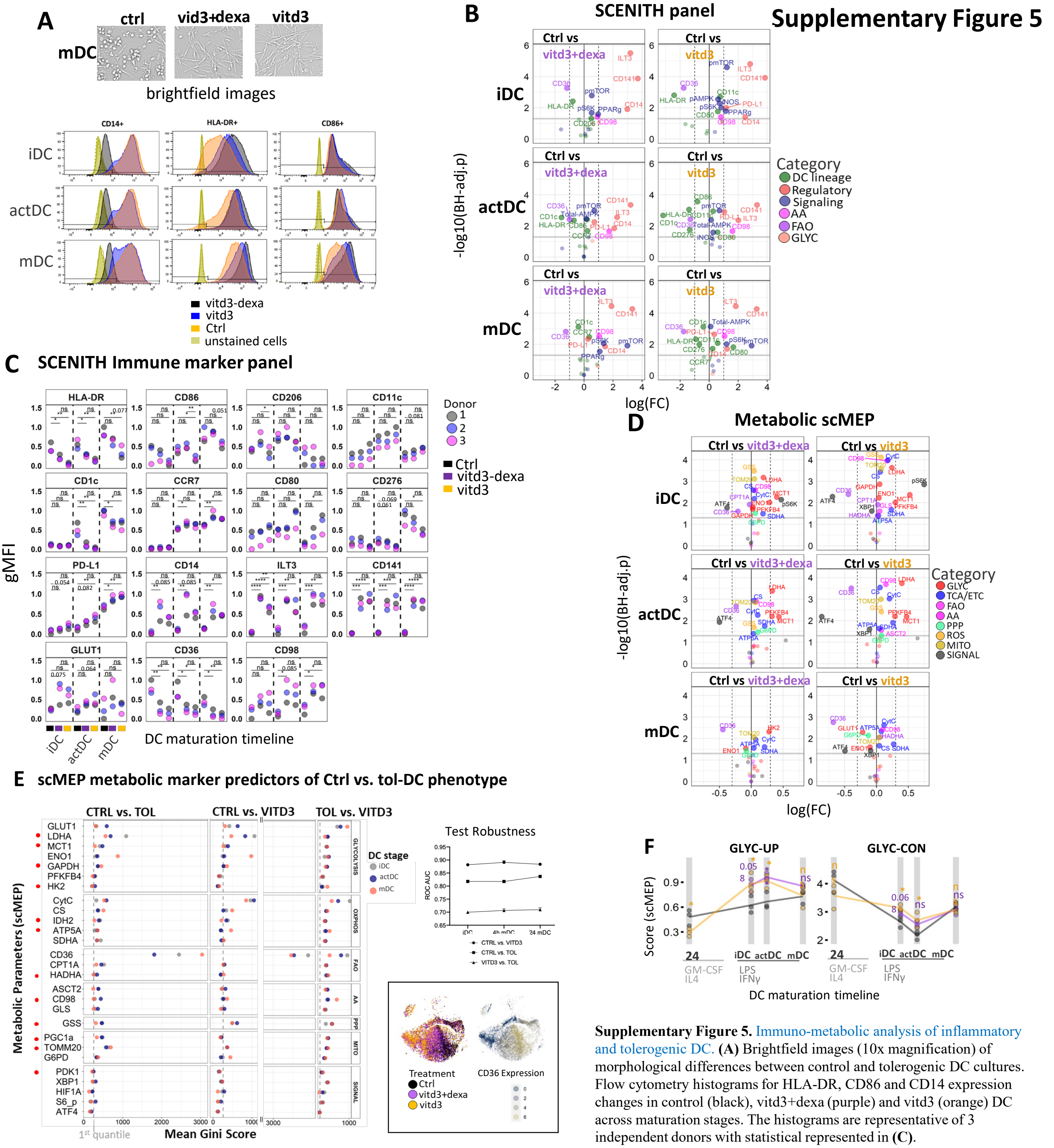

Supplementary Figure 5. Immuno-metabolic analysis of inflammatory and tolerogenic DC. **(A)** Brightfield images (10x magnification) of morphological differences between control and tolerogenic DC cultures. Flow cytometry histograms for HLA-DR, CD86 and CD14 expression changes in control (black), vitd3+dexa (purple) and vitd3 (orange) DC across maturation stages. The histograms are representative of 3 independent donors with statistical represented in **(C)**.

(B) Volcano plots show differential gMFI SCENITH marker expression by fold change (logFC) and BH FDR adjusted p-value (-log10(BH-adj.p)) comparing vitd3+dexa and vitd3 treated cells from controls at iDC, actDC, and mDC stages (N = 3 donors). Colors represent functional categories of the profiled markers. For orientation purposes, horizontal solid lines indicate significance threshold (BH-adj.p = 0.05) with vertical dotted lines marking fold change ≤ 1.5. **(C)** gMFI expression values of SCENITH panel surface receptor profiles in control (black), vitd3+dexa (purple) and vitd3 (orange) treatments across maturation stages for three (color-coded) donors. Statistical Significance was calculated using one-way ANOVA with Tukey's *post-hoc* test. **(D)** Volcano plots show differential median scMEP marker expression by fold change (logFC) and BH FDR adjusted p-value (-log10(BH-adj.p)) comparing vitd3+dexa and vitd3 treated cells from controls at iDC, actDC, and mDC stages (N = 3 donors). Colors indicate respective scMEP metabolic pathways. For orientation purposes, horizontal solid lines indicate significance threshold (BH-adj.p = 0.05) with vertical dotted lines marking fold change ≤ 1.5. **(E)** Calculated Gini impurity scores determining the relative importance of metabolic markers discriminating control and tolerogenic-treatments across DC maturation stages. Single-cell expression data were randomly divided into training and validation groups and metabolic scMEP parameters were used in the random forest model testing. Resulting area under the receiver operating characteristic curves (AUC-ROC) indicates the effectiveness of model performance. Comparative tSNE visualization of clustering similarities treatment conditions and CD36 single-cell heatmap overlays. **(F)** Kinetic profiles for calculated median scMEP pathways scores for control (black), vitd3+dexa (purple) and vitd3 (orange)-treated DC across DC maturation timeline. Connecting lines visualize mean pathway changes (N = 3). Statistical significance of pairwise comparisons between control and vitd3-dexa-tol (purple asterisk) and control and vitd3-tol samples (orange asterisk) analyses are depicted.

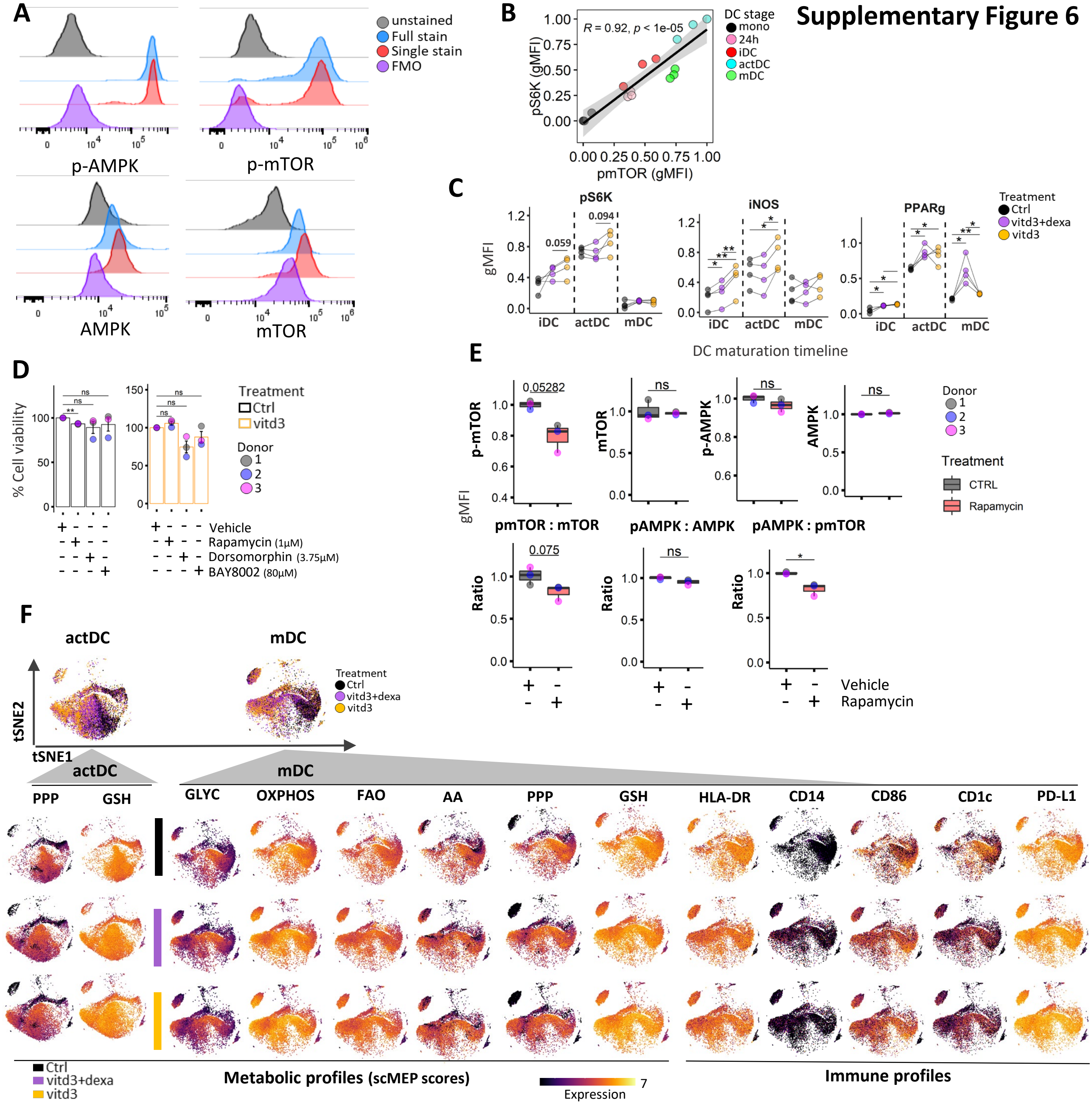

Supplementary Figure 6. Immuno-metabolic profiling of stochastic heterogeneity in control and tolerogenic DC. **(A)** Flow cytometry histograms for validation of specificity for antibodies detecting p-AMPK, total AMPK (AMPK), p-mTOR and total mTOR (mTOR) in fully-stained, single stained and fully-stained minus antibody of interest mature DC samples. Unstained controls are included for comparative purposes. **(B)** Correlation between gMFO of p-mTOR and pS6K across DC stages with Spearman correlation coefficient (R) and grey shading denoting 95% confidence interval (CI) are shown (N = 3 donors). **(C)** gMFI expression values of pS6K, iNOS and PPARγ in control (black), vitd3+dexa (purple) and vitd3 (orange)-treated DC across maturation stages are shown (N = 4). Lines connect data points from an individual donor. **(D)** Bar graphs with mean±SE represent % viability frequencies in of DC cultured in the presence of Vehicle (DMSO), Rapamycin (1 μM), Dorsomorphin (3.75 μM) and BAY8002 (80 μM) (N = 3). **(E)** box plots represent gMFI expression/phosphorylation of signaling factors and their indicated calculated rations in Control and Rapamycin (1 μM) samples treated with LPS/IFNγ for 30 minutes from 3 independent donors. **(F)** tSNE analyses based on metabolic marker expression of control (black), vitd3+dexa (purple) and vitd3 (orange)-treated DC across 4h and 24h maturation stages from one donor (out of N = 3 donors) are shown. Heatmap overlay of single-cell scMEP metabolic pathway scores and expression of phenotyping markers are depicted at actDC, and mDC stage to emphasize both immune and underlying metabolic heterogeneity as well as differences between control and tolerogenic DC. **(C-E)** Statistical Significance was calculated using one-way ANOVA with Tukey's *post-hoc* test.

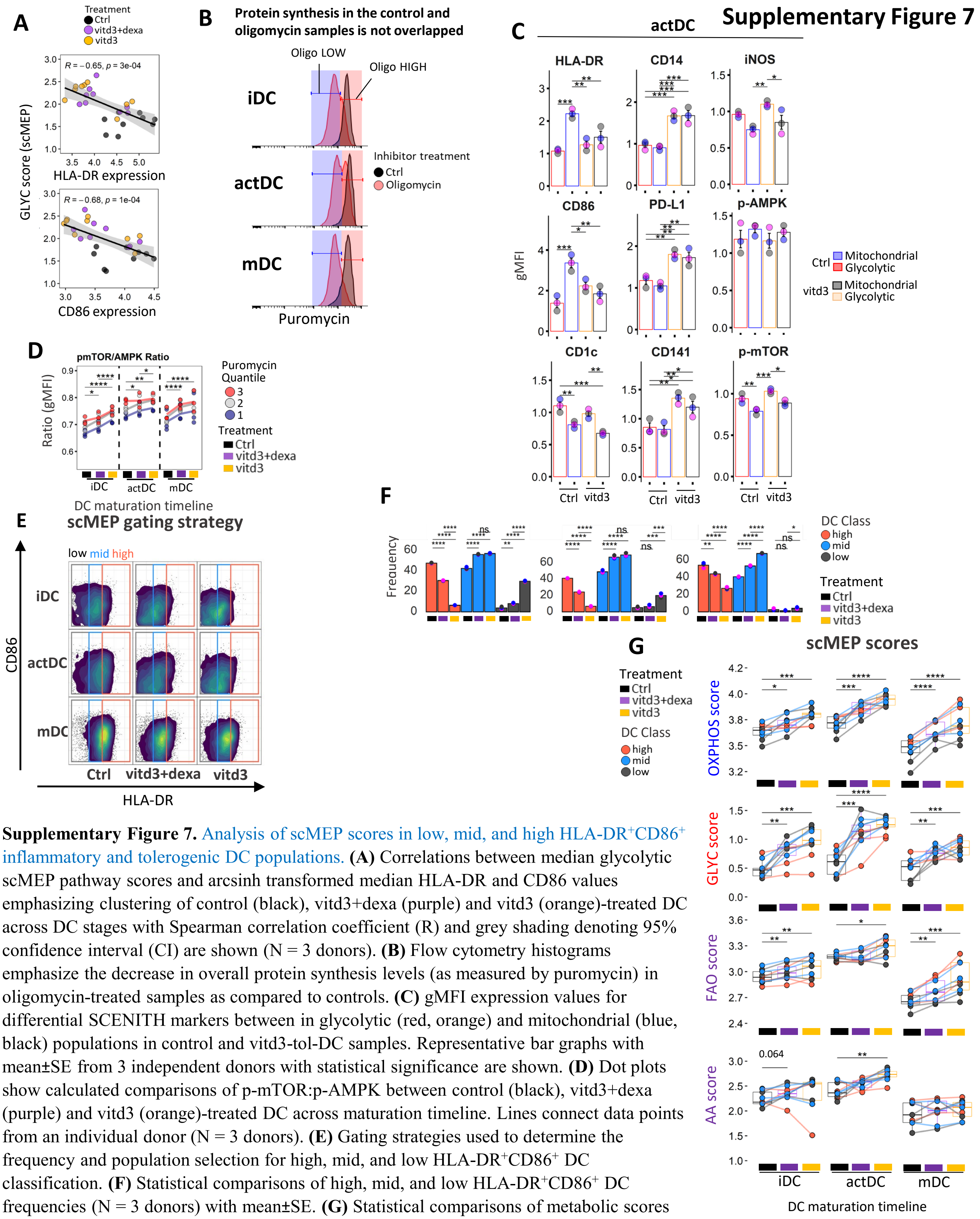

Supplementary Figure 7. Analysis of scMEP scores in low, mid, and high HLA-DR$^+$CD86$^+$ inflammatory and tolerogenic DC populations. **(A)** Correlations between median glycolytic scMEP pathway scores and arcsinh transformed median HLA-DR and CD86 values emphasizing clustering of control (black), vitd3+dexa (purple) and vitd3 (orange)-treated DC across DC stages with Spearman correlation coefficient (R) and grey shading denoting 95% confidence interval (CI) are shown (N = 3 donors). **(B)** Flow cytometry histograms emphasize the decrease in overall protein synthesis levels (as measured by puromycin) in oligomycin-treated samples as compared to controls. **(C)** gMFI expression values for differential SCENITH markers between in glycolytic (red, orange) and mitochondrial (blue, black) populations in control and vitd3-tol-DC samples. Representative bar graphs with mean±SE from 3 independent donors with statistical significance are shown. **(D)** Dot plots show calculated comparisons of p-mTOR:p-AMPK between control (black), vitd3+dexa (purple) and vitd3 (orange)-treated DC across maturation timeline. Lines connect data points from an individual donor (N = 3 donors). **(E)** Gating strategies used to determine the frequency and population selection for high, mid, and low HLA-DR$^+$CD86$^+$ DC classification. **(F)** Statistical comparisons of high, mid, and low HLA-DR$^+$CD86$^+$ DC frequencies (N = 3 donors) with mean±SE. **(G)** Statistical comparisons of metabolic scores levels in HLA-DR$^+$CD86$^+$ high, mid, and low DC populations between control and tolerogenic treatments across differentiation states (N = 3 donors). Lines connect data points from an individual donor. **(C-D, F-G)** Statistical Significance was calculated using one-way ANOVA with Tukey's *post-hoc* test.

Supplementary Table 1. SCENITH panel and antibody information

Marker	Fluorophore	Clone	Host	Company	Catalogue #
**Antibodies used in matured DC SCENITH profiling panel**					
CD14	BUV395	MϕP9	Mouse	BD	563561
AMPK	Dyligtht 350	2B7	Mouse	Novus Bio	NBP2-22127UV
CD276	BUV496	7-517	Mouse	BD	749897
CD274	BUV563	MIH1	Mouse	BD	741423
CD303	BUV615	V24-785	Mouse	BD	751078
HLA-DR	BUV805	G46-6	Mouse	BD	748338
CD1c	BV421	L161	Mouse	BioLegend	331525
iNOS	eFluor 450	CXNFT	Rat	eBioscience/TF	48-5920-80
CCR7	BV480	3D12	Rat	BD	566170
CD80	BV510	2D10	Mouse	BioLegend	305233
CD141	BV605	M80	Mouse	BioLegend	344117
CD206	BV711	15-2	Mouse	BioLegend	321135
ILT-3	BV750	ZM3.8	Mouse	BD	747371
CD86	BV785	IT2.2	Mouse	BioLegend	305441
anti-Puromycin	AF488	12D10	Mouse	Millipore Sigma	MABE343-AF488
CD11c	PerCP	BU15	Mouse	Thermo Fisher	A15803
CD273	BB700	MIH18	Mouse	BD	746072
CD36	PE	5-271	Mouse	BioLegend	336206
p-mTOR1	PE	O21-404	Mouse	BD	563489
PPARg	AF594	NA	Rat	Bioss Inc	bs-4590R-A594
pS6K	PE-Cy5.5	OTI1G4	Mouse	Novus	NBP2-73209PECY55
p-mTOR1 (Ser 2448)	PE-Cy7	MRRBY	Mouse	eBioscience/TF	50-112-3458
CD98	PE-Vio770	REA387	Rat	Myltenyi Biotec	130-126-200
-AMPKa-1/2 (Thr183/Thr172	AF647	NA	Rat	Bioss Inc	bs-4002R-A647
Zombie NIR Fixable Viability Kit				BioLegend	423105
GLUT1	APC/Cy7	not specified	Rat	Bioss Inc	SPC-1295D-APCCY7
**Antibodies used in fresh PBMC SCENITH profiling panel**					
FceRI	BUV805	AER-37	Mouse	BD	749337
CD14	BV605	63D3	Mouse	Bioloegend	367125
CD16	BV785	3G8	Mouse	Bioloegend	302045
CD11c	PE-Cy5	B-ly6	Mouse	BD	551077
CD206	BV711	15.2	Mouse	Bioloegend	321135
HLA-DR	BV650	L243	Mouse	Bioloegend	307649
CD141 (BDCA-3)	BV480	1A4	Mouse	BD	746604
CD3	lovaFluor Blue 61	UCHT1	Mouse	eBioscience/TF	H002T02B05
CD88	APC-Fire 750	S5/1	Mouse	Bioloegend	344315
CD89	APC-Fire 750	A59	Mouse	Bioloegend	354115
CD56	Alexa Fluor 532	NCAM1/795	Mouse	Novus Biological	NBP2-47826AF532
CD19	V450	HIB19	Mouse	BD	560353
CD123	BUV496	6H6	Mouse	BD	751836
CD45RA	BV570	HI100	Mouse	Bioloegend	304132
CD1c	BV421	L161	Mouse	Bioloegend	331526

Listed are protein targets used in conjunction with SCENITH functional metabolic profiling of DC differentiation states.

Supplementary Table 2. scMEP panel and antibody information

Metal	Myeloid/DC	clone	Column2	Group
89	CD45	HI30	surface	DC
113	CD11c	Bu15	surface	DC
115	CD11b	ICRF44	surface	DC
127	IdU		other	DNA_RNA_PROT
140	CD3	UCHT1	surface	DC
141	CD98	UM7F8	surface	AA
142	HADHA	EPR17940	intracellular	FAO
143	GSS	EPR6563	intracellular	ROS
144	XBP1	polyclonal	intracellular	SIGNAL
145	GLS	polyclonal	intracellular	AA
146	ATF4	EPR18111	intracellular	SIGNAL
147	GAPDH	6C5	intracellular	GLYC
148	CD14	RMO52	surface	DC
149	CytC	6H2.B4	intracellular	ETC_TCA
150	SDHA	2E3GC12FB2AE2	intracellular	ETC_TCA
151	Puromycin	12D10	intracellular	DNA_RNA_PROT
152	ENO1	EPR10863(B)	intracellular	GLYC
153	CS	EPR8067	intracellular	ETC_TCA
154	BrU	3D4	intracellular	DNA_RNA_PROT
155	CD163	GHI/61	surface	HEME
156	PFKFB4	polyclonal	intracellular	GLYC
157	PDK1	2H3AA11	intracellular	SIGNAL
158	ATP5A	15H4C4	intracellular	ETC_TCA
159	CD86	IT2.2	surface	DC
161	TOMM20	EPR15581-54	intracellular	MITO
162	G6PD	EPR20668	intracellular	PPP
163	CD36	5-271	surface	FAO
164	CD1c	L161	surface	DC
165	PGC1a	polyclonal	intracellular	MITO
166	GLUT1	EPR3915	surface	GLYC
167	CD303	201A	surface	DC
168	CD206	15-2	surface	DC
169	LDHA	EP1566Y	intracellular	GLYC
170	IDH2	EPR7577	intracellular	ETC_TCA
171	HK2	3D3	intracellular	GLYC
172	MCT1	P14612	surface	GLYC
173	CPT1A	8F6AE9	intracellular	FAO
174	ASCT2	CAL33	surface	AA
175	PDL1	29E.2A3	surface	DC
176	HIF1A	EP1215Y	intracellular	SIGNAL
196	S6_p	A17020B	intracellular	SIGNAL
198	dead			
209	HLA-DR	L243	surface	DC

Listed are metal conjugates and protein targets used in scMEP profiling.
