# Supplementary material for: Distinct metabolic states guide maturation of inflammatory and tolerogenic dendritic cells
Source: Nat Commun. 2022 Sep 2;13:5184. doi: 10.1038/s41467-022-32849-1 (PMC9440236; doi:10.1038/s41467-022-32849-1)
Supplement: Supplementary file 2 — Reporting Summary [file 41467_2022_32849_MOESM2_ESM.pdf]

nature portfolio

Corresponding author(s): Lisa H Butterfield

Last updated by author(s): 7/8/2022

Reporting Summary

Nature Portfolio wishes to improve the reproducibility of the work that we publish. This form provides structure for consistency and transparency in reporting. For further information on Nature Portfolio policies, see our Editorial Policies and the Editorial Policy Checklist.

Statistics

For all statistical analyses, confirm that the following items are present in the figure legend, table legend, main text, or Methods section.

n/a	Confirmed	
☐	☒	The exact sample size (*n*) for each experimental group/condition, given as a discrete number and unit of measurement
☐	☒	A statement on whether measurements were taken from distinct samples or whether the same sample was measured repeatedly
☐	☒	The statistical test(s) used AND whether they are one- or two-sided<br>*Only common tests should be described solely by name; describe more complex techniques in the Methods section.*
☐	☒	A description of all covariates tested
☐	☒	A description of any assumptions or corrections, such as tests of normality and adjustment for multiple comparisons
☐	☒	A full description of the statistical parameters including central tendency (e.g. means) or other basic estimates (e.g. regression coefficient) AND variation (e.g. standard deviation) or associated estimates of uncertainty (e.g. confidence intervals)
☐	☒	For null hypothesis testing, the test statistic (e.g. *F*, *t*, *r*) with confidence intervals, effect sizes, degrees of freedom and *P* value noted<br>*Give P values as exact values whenever suitable.*
☒	☐	For Bayesian analysis, information on the choice of priors and Markov chain Monte Carlo settings
☐	☒	For hierarchical and complex designs, identification of the appropriate level for tests and full reporting of outcomes
☐	☒	Estimates of effect sizes (e.g. Cohen's *d*, Pearson's *r*), indicating how they were calculated

Our web collection on statistics for biologists contains articles on many of the points above.

Software and code

Policy information about availability of computer code

Data collection	FACs data was acquired on the Cytek Aurora flow cytometer using the SpectroFlo software (v2.2.0.1, Cytek). Cells were also acquired on a CyTOF2 mass cytometer (Fluidigm).
Data analysis	FACs samples were unmixed using reference controls generated in combination with stained Ultracomp beads (Fisher Scientific, 01-2222-41) and stained cells using the SpectroFlo Software v2.2.0.1. The unmixed FCS files were used for data processing and analysis using FlowJo (BD, version 10.8.1) and CellEngine (CellCarta). Manually gated CD14-HLA-DR+CD86+ cells were used for downstream analysis using R (version 4.0.2). Raw mass spectrometry data were pre-processed, de-barcoded and imported into R environment using the flowCore package (version 2.0.1) (Hahne et al., 2009). Values were arcsinh transformed (cofactor 5) and normalized (Hartmann et al., 2021) for downstream analyses based on previously reported workflow (Nowicka et al., 2017). Mean cell radius (forward scatter from Cytek analysis, FSC-A) was used to calculate changes in cell volume across DC differentiation. Expression of scMEP factors was normalized to account for increase in cell volume from precursors to mature DC. All clustering analyses were performed on subsampled (20-25,000 cells/treatment time point) HLA-DR+CD86+-gated cell populations with indicated input markers. Multi-dimensional plots were generated using R package limma (version 3.44.) and dimensionality reduction analysis was performed using Rtsne (version 0.15) Uniform Manifold Approximation and Projection (UMAP) was performed using FlowJo. For visualization and heatmap clustering we utilized R packages ggplot2 (version 3.3.3) and ComplexHeatmap (version 2.4.3) (Gu et al., 2016), respectively. Differential expression analysis of marker expression between treatment groups was determined separately for each DC maturation time point using linear mixed effect model accounting for donor variability using the lme4 (version 1.1-26) package. Spearman correlation coefficient correlation matrix for marker expression profiles was computed and visualized using the corrr (version 0.4.3), Hmisc (version 4.5.0) and corrplot (version 0.88) R packages.To determine OXPHOS, glycolysis, FAO and AA scMEP pathway scores we applied linear regression analysis between the scMEP median metabolic marker expression (arcsinh transformed) and log-transformed median SCENITH parameters (adjusted to protein synthesis) using the R package lmerTest (version 3.13). For calculating scMEP scores, the most significant and positively correlated markers within each metabolic pathway were summarized and divided by the number of markers within that pathway. Spearman correlations between scMEP pathway scores and SCENITH parameters were represented using the ggpubr (version, 0.4.0) R package. Random forests were performed using the package randomForest (version, 4.6 – 14). Statistical testing was

conducted using the rstatix (version 0.7.0) with graphs and significance labels generated using ggplot2 (version 3.3.3) and ggpubr (0.4.0) respectively.

For manuscripts utilizing custom algorithms or software that are central to the research but not yet described in published literature, software must be made available to editors and reviewers. We strongly encourage code deposition in a community repository (e.g. GitHub). See the Nature Portfolio guidelines for submitting code & software for further information.

Data

Policy information about availability of data

All manuscripts must include a data availability statement. This statement should provide the following information, where applicable:

- Accession codes, unique identifiers, or web links for publicly available datasets
- A description of any restrictions on data availability
- For clinical datasets or third party data, please ensure that the statement adheres to our policy

The datasets generated during and/or analyzed during the current study are available from the corresponding author on reasonable request.

Field-specific reporting

Please select the one below that is the best fit for your research. If you are not sure, read the appropriate sections before making your selection.

☒ Life sciences ☐ Behavioural & social sciences ☐ Ecological, evolutionary & environmental sciences

For a reference copy of the document with all sections, see nature.com/documents/nr-reporting-summary-flat.pdf

Life sciences study design

All studies must disclose on these points even when the disclosure is negative.

Sample size	For all experiments n = 3 was chosen as the minimal independent replicate number with proper internal controls. For dimensionality reduction we used 20-25,000 cells, which was sufficient for all downstream analyses and significance testing.
Data exclusions	Data were not excluded from analysis.
Replication	Independent experimental replicates (from multiple donors as well as technical replicates) were conducted to verify data reproducibility.
Randomization	All replication attempts were successful and observed marker expression profiles were consistent with orthogonal methods and previously published results. The study was performed on isolated PBMCs from participants, which were randomly allocated from the supplier. Covariates are not relevant, as the samples were obtained from healthy donors. Sample and replicate numbers are included in the figure legends
Blinding	Blinding was not possible and anonymous healthy donors were the sources of cells used. There are no mouse treatment or human treatment groups to compare. In vitro experiments can't be blinded and have accurate metabolic inhibitors added and be antibody stained and analyzed.

Reporting for specific materials, systems and methods

We require information from authors about some types of materials, experimental systems and methods used in many studies. Here, indicate whether each material, system or method listed is relevant to your study. If you are not sure if a list item applies to your research, read the appropriate section before selecting a response.

Materials & experimental systems

n/a	Involved in the study	
☐	☒	Antibodies
☒	☐	Eukaryotic cell lines
☒	☐	Palaeontology and archaeology
☒	☐	Animals and other organisms
☐	☒	Human research participants
☒	☐	Clinical data
☒	☐	Dual use research of concern

Methods

n/a	Involved in the study	
☒	☐	ChIP-seq
☐	☒	Flow cytometry
☒	☐	MRI-based neuroimaging

Antibodies

Antibodies used	Fluorescently-conjugated antibodies used millipore SIgma in the study include: CD14 (BD, 563561), AMPK (Novus Bio, NBP2-22127UV), CD276 (BD, 749897), CD274 (BD, 741423), CD303 (BD, 751078), HLA-DR (BD, 748338), CD1c (BioLeged, 331525), iNOS (eBioscience, 48-5920-80), CCR7 (BioLegend, 331525), CD80 (BioLeged, 305233), CD141 (BioLeged, 344117), CD206 (BioLeged,

321135), ILT-3 (BD, 747371), CD86 (BioLeged, 305441), anti-Puro (Millipore Sigma, MABE343-AF488, clone 12D10), CD11c (Thermo Fisher, A15803), CD273 (BD, 746072), CD36 (BioLeged, 336206), p-mTOR1 (BD, 563489), PPARg (Bioss Inc, bs-4590R-A594), pS6K (NBP2-73209PECY55,Novus Bio), p-mTOR1 (Ser 2448) (eBioscience, 50-112-3458), CD98 (130-126-200, Myltenyi Biotec), p-AMPKa-1/2 (Thr183/Thr172) (Bioss Inc, bs-4002R-A647), GLUT1 (StressMarq, SPC-1295D-APCCY7), FceRI (BD, 749337), CD11c (BD, 551077), CD3 (eBioscience/TF, H002T02B05), CD88 (Bioloegend,344315), CD89 (Bioloegend, 354115) CD56 (Novus Bio, NBP2-47826AF532), CD19 (BD, 560353), CD123 (BD, 751836), CD45RA (Bioloegend, 304132)

Validation

Biolegend, Novus Bio, Thermo Fisher/eBioscience, MilliporeSigma and Bioss antibodies were tested in a variety of assays and each selected antibody clone suitable for flow cytometry applications recognizing human protein targets was tested on models designed to carefully assess the desired specificity and sensitivity of the antibodies. scMEP antibodies targeting metabolic features were conjugated in-house using an optimized conjugation protocol (Hartmann et al., 2019) and validated on multiple sample types.

Human research participants

Policy information about studies involving human research participants

Population characteristics	Supplier provided samples from healthy human donors of different age and sex.
Recruitment	Healthy donors from Vitalant Research Institute (San Francisco, CA).
Ethics oversight	IRB-approved protocol in Denver, CO and San Francisco, CA.

Note that full information on the approval of the study protocol must also be provided in the manuscript.

Flow Cytometry

Plots

Confirm that:

☒ The axis labels state the marker and fluorochrome used (e.g. CD4-FITC).

☒ The axis scales are clearly visible. Include numbers along axes only for bottom left plot of group (a 'group' is an analysis of identical markers).

☒ All plots are contour plots with outliers or pseudocolor plots.

☒ A numerical value for number of cells or percentage (with statistics) is provided.

Methodology

Sample preparation	PBMCs from healthy donors were purchased (Trima Residuals RE202, Vitalant) and purified by Ficoll-hypaque gradient centrifugation (Fisher Scientific, 45-001-749). Cryopreserved PBMCs were thawed using RPMI (Gibco-Invitrogen) complete media (1% Pen Strep, 1% L-Glutamine, 10% FBS Heat Inactivated Serum (Gibco-Invitrogen, 16000-044), and 0.5% DNase (Sigma, DN-25) and washed twice with PBS. CD14+ monocytes were selected using CD14 microbeads (Miltenyi Biotec, 130-050-201) and cultured for 5 days in CellGenix medium (0020801-0500) supplemented with 800 U/mL GM-CSF (Miltenyi Biotec, 130-095-372) and 500 U/mL IL4 (Miltenyi Biotec, 130-095-373) to generate iDC. At day 3, half of media was replaced and supplemented with fresh cytokines. iDC were matured on day 5 with 1000 U/mL IFN- γ (Peprotech, 300-02) and 250 ng/mL LPS (Sigma-Aldrich, L2630). Two types of tol-moDC were generated. To obtain vitd3-tol-moDC 100nM of vitamin D3 (Sigma, D1530) was added to cultures at d0 and day3. And dexa-vitd3-tol-moDC were generated by adding 100nM of vitamin D3 and 10 nM of dexamethasone (Sigma, D4902) at day 3 to cultures. Both tol-moDC were matured as described above.
Instrument	Samples were acquired on the Aurora flow cytometer (Cytek). scMEP data was acquired on a CyTOF2 mass cytometer (Fluidigm).
Software	SpectroFlo Software v2.2.0.1. and FlowJo (BD, version 10.7.1).
Cell population abundance	CD14+ monocytes were selected using CD14 microbeads (Miltenyi Biotec, 130-050-201) according to manufacturer instructions, viability of samples was analyzed using the Cellometer Spectrum (Nexcelom Biosciences).
Gating strategy	Gating strategies used to determine frequencies and early precursor stages for CD14+ monocyte (top), 24h post-GM-CSF/IL4 stimulus CD14+ HLA-DRLo (middle) and matured moDC HLA-DR+CD86+ populations (bottom) are indicated in supplemental figure 1. Initially, single cells were identified based on SSC-A, FSC-H, and FSC-A, FSC-H scatter. Live/Dead (Zombie NIR) dye as well as puromycin staining were used to select live cell populations.

☒ Tick this box to confirm that a figure exemplifying the gating strategy is provided in the Supplementary Information.
